# Intranasal kisspeptin administration rapidly stimulates gonadotropin release in humans

**DOI:** 10.1016/j.ebiom.2025.105689

**Published:** 2025-04-11

**Authors:** Edouard G. Mills, Mauro S.B. Silva, Virginia Delli, Laurine Decoster, Gaetan Ternier, Jovanna Tsoutsouki, Layla Thurston, Maria Phylactou, Bijal Patel, Lisa Yang, Sophie A. Clarke, Megan Young, Emma C. Alexander, Sandhi Nyunt, Arthur C. Yeung, Muhammad Choudhury, Anastasia Newman, Paul Bech, Ali Abbara, Magda Swedrowska, Ben Forbes, Vincent Prévot, Konstantina Chachlaki, Paolo Giacobini, Alexander N. Comninos, Waljit S. Dhillo

**Affiliations:** a Section of Endocrinology and Investigative Medicine, Imperial College London, London, UK; b Department of Endocrinology, Imperial College Healthcare NHS Trust, London, UK; c Laboratory of Development and Plasticity of the Neuroendocrine Brain, Lille Neuroscience and Cognition, Univ. Lille, Inserm, CHU Lille, Lille, France; d Institute of Pharmaceutical Science, King’s College London, London, UK

**Keywords:** Kisspeptin, Fertility, Neuropeptides, Reproduction, Neuroendocrinology

## Abstract

**Background:**

Kisspeptin administration by *intravenous* or *subcutaneous* routes activates hypothalamic gonadotropin-releasing hormone (GnRH) neurons and is being developed to treat reproductive disorders. However, these invasive routes markedly limit patient acceptability and clinical use. Recent rodent data has identified a large GnRH population within the olfactory system communicating directly with hypothalamic GnRH neurons. *Intranasal* kisspeptin administration may be able to capitalise on this novel pathway and thus offer a potential non-invasive approach to stimulate reproductive hormones. Herein, we examine *intranasal* kisspeptin using human, pharmaceutical, and rodent studies.

**Methods:**

Reproductive hormone profiles were measured after intranasal kisspeptin administration in healthy volunteers and patients with reproductive disorders as part of a randomised, double-blinded, crossover, placebo-controlled clinical study. Pharmaceutical testing evaluated the chemical stability and nasal kisspeptin delivery, and rodent studies provided mechanistic insight.

**Findings:**

Intranasal kisspeptin-54 rapidly stimulates gonadotropin release in healthy men and women, and in patients with a common reproductive disorder (hypothalamic amenorrhoea), without any side effects or adverse events encountered. Specifically, intranasal kisspeptin (at 12.8 nmol/kg) induced clinically-significant mean maximal increases above baseline in serum luteinising hormone in all study groups: 4.4 ± 0.6 IU/L (mean difference = 3.1 IU/L [95% CI, 1.2–4.9], *P* = 0.002 vs. placebo) in healthy men; 1.4 ± 0.3 IU/L (mean difference = 1.0 IU/L [95% CI, 0.4–1.7], *P* = 0.004 vs. placebo) in healthy women; 4.4 ± 0.2 IU/L (mean difference = 4.3 IU/L [95% CI, 2.7–6.0], *P* < 0.001 vs. placebo) in patients with hypothalamic amenorrhoea. Kisspeptin-54 was delivered effectively via nasal spray and was stable for up to 60 days at 4 °C. Mirroring the human effects, intranasal kisspeptin-54 in adult C57BL/6J male mice stimulates luteinising hormone release. Further mechanistic insights reveal the accumulation of fluorescently-tagged kisspeptin in the olfactory epithelium, as well as the presence of kisspeptin receptors in olfactory bulb GnRH neurons, implicating the involvement of these extra-hypothalamic GnRH neurons in the pathway mediating intranasal kisspeptin’s effects on reproductive hormones.

**Interpretation:**

We demonstrate the clinical potential for intranasal kisspeptin delivery as the first non-invasive method to robustly and safely stimulate gonadotropins with kisspeptin and potentially transform the management of reproductive disorders.

**Funding:**

10.13039/501100000272National Institute for Health and Care Research (NIHR)/10.13039/501100013342NIHR Imperial Biomedical Research Centre/10.13039/501100000265Medical Research Council (MRC).

**Research in context**

**Evidence before this study**

The neuropeptide kisspeptin is a critical endogenous activator of the reproductive system. Due to its key role in regulating reproductive processes, there has been escalating clinical interest in targeting kisspeptin-pathways to treat common reproductive disorders, as well as metabolic, psychosexual, and bone disorders. However, administration of kisspeptin is currently confined to the invasive subcutaneous or intravenous routes to access *hypothalamic* gonadotropin-releasing hormone (GnRH) neurons, which present barriers for patient and clinical acceptability.

It is notable that recent rodent data has identified a large GnRH population within the olfactory bulb (OB), which extends neurites into the olfactory epithelium and projects to the hypothalamic preoptic area and to the median eminence, thereby connecting olfactory cues with the reproductive endocrine axis. Kisspeptin is an established activator of *hypothalamic* GnRH neurons, but whether kisspeptin can also exploit this recently characterised *extra-hypothalamic* GnRH population to stimulate downstream reproductive function is currently unknown. Such activation would provide the basis for a novel non-invasive treatment approach for treating patients with reproductive disorders and therefore be potentially practice-changing.

**Added value of this study**

Using a series of human, pharmaceutical, and rodents studies, we examine the therapeutic potential of intranasal kisspeptin administration for the first time. We demonstrate that intranasal kisspeptin administration can rapidly stimulate gonadotropin release in healthy men and women, and in patients with a common reproductive disorder (hypothalamic amenorrhoea), without any side effects or adverse events encountered. From a pharmaceutical development perspective, we demonstrate that kisspeptin in 0.9% saline solution remains within pharmaceutically accepted limits for stability for up to 60 days at 4 °C (i.e., typical home refrigerator temperature), providing a realistic opportunity to create a patient self-administered nasal medicine with significant therapeutic benefits. Finally, consistent with the effects observed in humans, intranasal kisspeptin in mice stimulates luteinising hormone release. Further mechanistic insights reveal the accumulation of fluorescently-tagged kisspeptin in the olfactory epithelium, as well as the presence of kisspeptin receptors in OB GnRH neurons, suggesting that these *extra-hypothalamic* GnRH neurons may be the direct targets of intranasal kisspeptin, driving its effects on gonadotropin release.

**Implications of all the available evidence**

Collectively, we provide the first human evidence identifying intranasal delivery as a novel, non-invasive, well-tolerated, and effective route of kisspeptin administration to safely stimulate gonadotropin release. This provides a realistic opportunity to create a patient self-administered nasal medicine with significant therapeutic benefits that would be far preferable to current invasive methods (injection) for patients and clinicians alike and so be practice-changing in the field.

## Introduction

The reproductive neuropeptide kisspeptin (encoded by the human *KISS1* gene) is a critical activator of gonadotropin-releasing hormone (GnRH) neurons in the hypothalamus, thereby stimulating downstream reproductive hormone release.^1^, ^2^, ^3^, ^4^, ^5^, ^6^, ^7^, ^8^ Clinical studies indicate that kisspeptin-based medicines have significant therapeutic potential to treat common reproductive disorders in humans, including hypothalamic amenorrhoea (HA),^4^^,^^9^, ^10^, ^11^ polycystic ovary syndrome,^4^ diabetes and obesity-related hypogonadism,^12^^,^^13^ hyperprolactinaemia,^14^^,^^15^ and are a safe and effective trigger for inducing oocyte maturation *in vitro* fertilisation therapy.^16^, ^17^, ^18^ Moreover, emerging evidence identifies kisspeptin as a potential therapeutic agent for human metabolic,^19^^,^^20^ psychosexual,^21^, ^22^, ^23^, ^24^, ^25^, ^26^, ^27^, ^28^ and bone disorders.^29^^,^^30^ However, current therapeutic application is confined to the subcutaneous or intravenous routes which limits patient acceptability and clinical use. Therefore, alternative delivery routes could overcome this and further accelerate the development of kisspeptin-based therapeutics in humans.

It is well-established that GnRH neurons originate outside the central nervous system in the medial olfactory placode during embryological development,^31^, ^32^, ^33^ from where they migrate towards the hypothalamus along an olfactory/vomeronasal axonal scaffold.^34^, ^35^, ^36^ However, curiously a notable extra-hypothalamic population of GnRH neurons persists within the olfactory bulbs (OB) of adult humans.^37^ Interestingly, very recent adult rodent data has identified that remarkably 20% of the entire brain population of GnRH neurons is located in the OB.^37^ Indeed, these extra-hypothalamic GnRH neurons extend neurites into the olfactory epithelium and project to the hypothalamic preoptic area and to the median eminence, connecting olfactory cues directly with the reproductive axis.^37^ Consistent with this, chemogenetic activation of OB GnRH neurons significantly increases the neuronal activity of GnRH neurons in the hypothalamic preoptic area followed by a strong luteinising hormone (LH) and testosterone secretion.^37^ Kisspeptin is an established activator of *hypothalamic* GnRH neurons, but whether kisspeptin can also activate this recently characterised *extra-hypothalamic olfactory* GnRH population to stimulate downstream reproductive function is currently unknown. Such activation would provide the basis for a novel non-invasive treatment approach for treating patients with reproductive disorders and therefore be potentially practice-changing.

Indeed, the delivery of peptide hormones via the nasal route has gained considerable attention with several under investigation, including insulin,^38^, ^39^, ^40^, ^41^ leptin,^42^, ^43^, ^44^ and oxytocin.^45^, ^46^, ^47^ Moreover, desmopressin administration via the intranasal route is favoured (over oral, subcutaneous, and intravenous) in the routine clinical endocrine treatment of patients with diabetes insipidus (arginine vasopressin deficiency).^48^ Therefore, there is a precedent for successful clinical intranasally-delivered treatments. However, there are currently no data from animal or human studies investigating the efficacy of intranasal kisspeptin delivery to stimulate reproductive hormone release.

Herein, we comprehensively examined intranasal kisspeptin administration for the first-time using human, pharmaceutical, and rodent studies. We first conducted human clinical studies to elucidate whether intranasal kisspeptin administration could indeed stimulate reproductive hormone secretion. Here, we evaluated the reproductive endocrine profiles after intranasal kisspeptin administration in healthy eugonadal participants, and crucially in a patient group of women with one of the commonest reproductive disorders, HA, accounting for 30% of cases of secondary amenorrhoea in women of reproductive age,^49^ where kisspeptin-based medicines may be a realistic therapy.^50^, ^51^, ^52^ Next, from a pharmaceutical development perspective, we characterised the performance of the nasal spray device and the chemical stability of kisspeptin in solution for nasal delivery. Finally, we undertook a series of rodent studies to provide mechanistic insight for how intranasal kisspeptin may be mediating these effects. Determining whether and how intranasal kisspeptin administration stimulates reproductive hormone release could potentially change future clinical practice by providing the first non-invasive, well-tolerated, and effective route of administration targeting kisspeptin-pathways to treat common reproductive disorders in humans.

## Methods

### Effects of intranasal administration of kisspeptin in healthy humans and patients with reproductive disorders

#### Ethics

Ethical approval for the clinical study conducted in healthy men was obtained from the Riverside Research Ethics Committee, London, UK (ref: 17/LO/1504) and the study in healthy women and patients with HA from the West London Research Ethics Committee, London, UK (ref: 12/LO/0507). The study was conducted in accordance with the Declaration of Helsinki and International Council for Harmonisation guidelines on Good Clinical Practice. The study was registered on the ISRCTN Registry (ref: 10095215) on 8th March 2022 with the first patient recruited on 3rd May 2022. The final patient completed the study on 10th May 2024. All participants provided written informed consent prior to participation.

#### Participants

Healthy men (aged ≥ 18 years), healthy ovulatory women (aged ≥ 18 years, menstrual cycle length less than 35 days, and not taking any hormonal therapies), and anovulatory women (aged ≥ 18 years and not taking hormonal therapies), were invited to take part through advertisements placed online and in local newspapers. Interested individuals underwent a detailed medical screening visit, including medical history, medication history, clinical examination, electrocardiogram, and blood tests. Participants were excluded based on the following criteria: history of medical or psychological conditions; use of prescription/recreational drugs in the preceding 6 months; use of an investigational drug in the preceding 2 months; pregnancy/breastfeeding; smoker; blood donation within 3 months of study participation. HA was diagnosed in accordance with Endocrine Society guidelines, which in brief requires excluding anatomical or organic causes of amenorrhoea in women whose menstrual cycle interval persistently exceeds 45 days and/or those who present with amenorrhoea for >3 months.^51^ To confirm health status, blood parameters were assessed at screening including full blood count, renal function, liver function, bone profile, thyroid hormone profile, LH, follicle-stimulating hormone (FSH), testosterone, and sex hormone-binding globulin. The following additional reproductive hormones were assessed in women: oestradiol, progesterone, androstenedione, dehydroepiandrosterone, anti-Müllerian hormone (AMH), and prolactin. Ethnicity data was collected through self-reported identification according to the UK Government classification system: (1) Asian, (2) Black, (3) Mixed or multiple ethnic groups, (4) White, and (5) Other ethnic group.^53^

Following screening and informed consent, 12 healthy men, 12 healthy women, and 10 patients with HA took part in the study, with all participants completing the protocol (including all study visits and no missing data) and therefore forming the dataset.

#### Study protocol

**Fig. 1:**
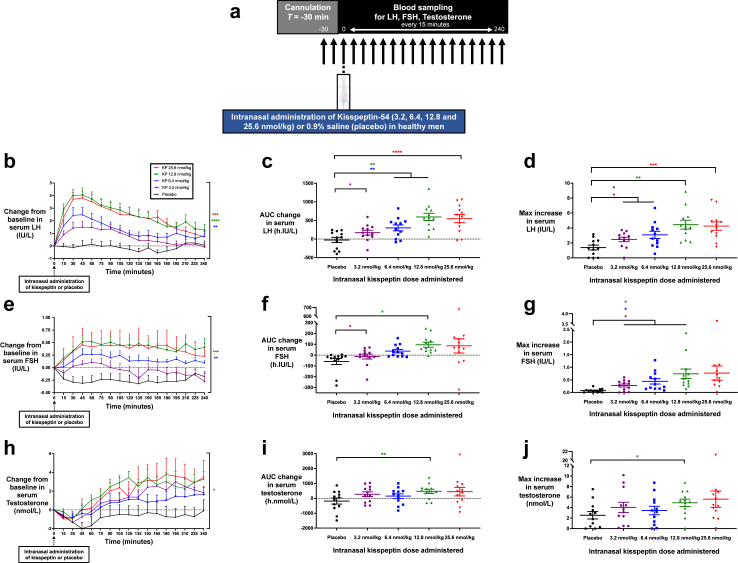
**Intranasal administration of kisspeptin stimulates reproductive hormone secretion in healthy men. (a)** Protocol schematic: Participants completed five study visits each, receiving the following five interventions via the intranasal route: 3.2, 6.4, 12.8, and 25.6 nmol/kg of kisspeptin-54, and 0.9% saline (placebo). After self-administration of kisspeptin-54 or placebo at timepoint 0 min, serum levels of LH, FSH, and testosterone were measured every 15 min for 4 h **(b, e and h)**: Mean (±SEM) change from baseline in serum LH (IU/L) **(b)**, serum FSH (IU/L) **(e)**, and serum testosterone (nmol/L) **(h)** in healthy men receiving intranasal administration of kisspeptin-54 or placebo. Groups were compared by two-way ANOVA with post-hoc Bonferroni multiple comparison test (asterisk denotes statistical significance for individual kisspeptin doses vs. placebo administration). **(c, f and i)**: Mean (±SEM) area under the curve (AUC) of the change in serum LH (h.IU/L) **(c)**, serum FSH (h.IU/L) **(f)**, and serum testosterone (h.nmol/L) **(i)** after intranasal administration of kisspeptin-54 or placebo in healthy men. Groups were compared by one-way ANOVA with post-hoc Bonferroni multiple comparison test (asterisk denotes statistical significance for individual kisspeptin doses vs. placebo administration). **(d, g and j)** Mean (±SEM) maximum increase from baseline in serum LH (IU/L) **(d)**, serum FSH (IU/L) **(g)**, and serum testosterone (nmol/L) **(j)** after intranasal administration of kisspeptin-54 or placebo in healthy men. Groups were compared by one-way ANOVA with post-hoc Bonferroni multiple comparison test (asterisk denotes statistical significance for individual kisspeptin doses vs. placebo administration). ∗*P* < 0.05, ∗∗*P* < 0.01, ∗∗∗*P* < 0.001, ∗∗∗∗*P* < 0.0001. *N* = 12.

**Fig. 2:**
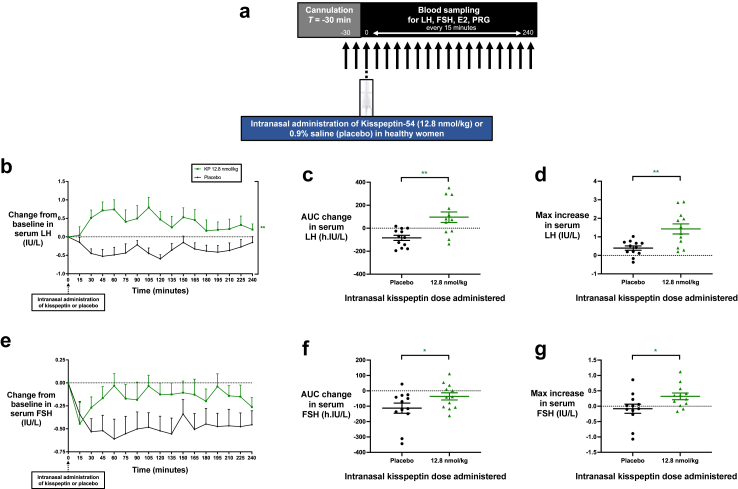
**Intranasal administration of kisspeptin stimulates reproductive hormone secretion in healthy women. (a)** Protocol schematic: Participants completed two study visits each, receiving 12.8 nmol/kg of kisspeptin-54 and placebo via the intranasal route. After self-administration of kisspeptin-54 or placebo at timepoint 0 min, serum levels of LH, FSH, oestradiol, and progesterone were measured every 15 min for 4 h. **(b and e)**: Mean (±SEM) change from baseline in serum LH (IU/L) **(b)** and serum FSH (IU/L) **(e)** in healthy women receiving intranasal administration of kisspeptin-54 or placebo. Groups were compared by two-way ANOVA with post-hoc Bonferroni multiple comparison test (asterisk denotes statistical significance for kisspeptin 12.8 nmol/kg vs. placebo administration). **(c and f)**: Mean (±SEM) area under the curve (AUC) of the change in serum LH (h.IU/L) **(c)** and serum FSH (h.IU/L) **(f)** after intranasal administration of kisspeptin-54 or placebo. Groups were compared by paired t-tests (asterisk denotes statistical significance for kisspeptin 12.8 nmol/kg vs. placebo administration). **(d and g)**: Mean (±SEM) maximum increase from baseline in serum LH (IU/L) **(d)** and serum FSH (IU/L) **(g)** after intranasal administration of kisspeptin-54 or placebo. Groups were compared by paired t-tests (asterisk denotes statistical significance for kisspeptin 12.8 nmol/kg vs. placebo administration). ∗*P* < 0.05, ∗∗*P* < 0.01. *N* = 12.

**Fig. 3:**
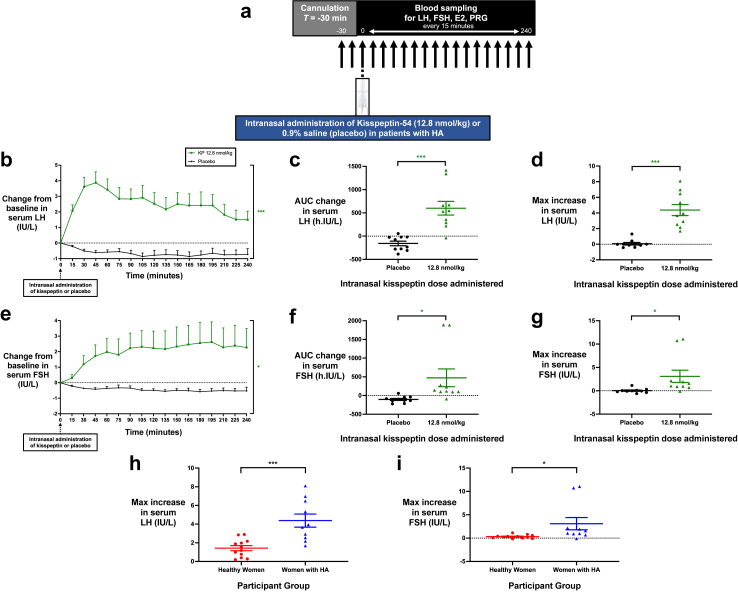
**Intranasal administration of kisspeptin stimulates reproductive hormone secretion in patients with hypothalamic amenorrhoea. (a)** Protocol schematic: Participants completed two study visits each, receiving 12.8 nmol/kg of kisspeptin-54 and placebo via the intranasal route. After self-administration of kisspeptin-54 or placebo at timepoint 0 min, serum levels of LH, FSH, oestradiol, and progesterone were measured every 15 min for 4 h. **(b and e)**: Mean (±SEM) change from baseline in serum LH (IU/L) **(b)** and serum FSH (IU/L) **(e)** in patients with HA receiving intranasal administration of kisspeptin-54 or placebo. Groups were compared by two-way ANOVA with post-hoc Bonferroni multiple comparison test (asterisk denotes statistical significance for kisspeptin 12.8 nmol/kg vs. placebo administration). **(c and f)**: Mean (±SEM) area under the curve (AUC) of the change in serum LH (h.IU/L) **(c)** and serum FSH (h.IU/L) **(f)** after intranasal administration of kisspeptin-54 or placebo. Groups were compared by paired t-tests (asterisk denotes statistical significance for kisspeptin 12.8 nmol/kg vs. placebo administration). **(d and g)**: Mean (±SEM) maximum increase from baseline in serum LH (IU/L) **(d)** and serum FSH (IU/L) **(g)** after intranasal administration of kisspeptin-54 or placebo. Groups were compared by paired t-tests (asterisk denotes statistical significance for kisspeptin 12.8 nmol/kg vs. placebo administration). **(h and i)**: Mean (±SEM) maximum increase from baseline in serum LH (IU/L) **(h)** and serum FSH (IU/L) **(i)** after intranasal administration of kisspeptin-54 at 12.8 nmol/kg in healthy women (presented in red) and women with hypothalamic amenorrhoea (presented in blue). Groups were compared by unpaired t-tests (asterisk denotes statistical significance for gonadotropin responses in healthy women vs. women with hypothalamic amenorrhoea). ∗*P* < 0.05, ∗∗∗*P* < 0.001. *N* = 10 (women with HA) and *N* = 12 (healthy women).

This was a randomised, double-blinded, crossover, placebo-controlled clinical study (Fig. 1, Fig. 2, Fig. 3a). Participants were admitted to the Clinical Research Unit (Imperial College Healthcare NHS Trust) and completed five (in healthy men) or two (in healthy women and patients with HA) study visits. To ensure washout, study visits were at least 1 week apart in healthy men and patients with HA, given that the circulating half-life of kisspeptin-54 is 27.6 min.^54^ Study visits in healthy women were conducted in the follicular phase of the menstrual cycle (i.e., days 2–10 inclusive) to control for changes in reproductive hormones over the course of the cycle and were conducted across two different menstrual cycles to allow washout.

All studies commenced at 08:00 to control for circadian hormonal changes. Participants were instructed to abstain from alcohol, caffeine, and sexual activities from 22:00 preceding each study visit. On arrival, an intravenous cannula was inserted into one antecubital fossa, and blood was sampled at timepoints −30, −15, and 0 min, before intranasal administration of kisspeptin-54 or 0.9% saline (placebo). Following participant education (described below), healthy men self-administered the following 5 interventions under supervision in random order via the intranasal route: 3.2, 6.4, 12.8, and 25.6 nmol/kg of kisspeptin-54, or placebo. Healthy women and patients with HA attended for the same protocol comparing 12.8 nmol/kg of kisspeptin-54 and placebo in random order. After administration of kisspeptin-54 or placebo at timepoint 0 min, serum levels of LH, FSH, and testosterone (in men), or oestradiol and progesterone (in women) were measured every 15 min for 4 h. Heart rate, blood pressure, and the presence of adverse symptoms were recorded every 15 min.

There were no changes to the study (including eligibility criteria, protocol, or outcome measures) after study commencement and no interim analysis. Data collection, storage, and monitoring were conducted by the authors with use of an electronic password-protected data-capture system and backed-up daily onto the Imperial College London server. The database lock occurred once all participants had completed the study protocol (sample size described below).

#### Randomisation

After informed consent and recruitment, participants were allocated an individualised participant number in sequential order. The participant numbers were coupled with an intervention sequence, with each participant receiving all interventions (i.e., five interventions for the healthy men, and two interventions for the healthy women and patients with HA). This was generated by author EGM prior to commencement of the study. For the study in healthy men, intervention sequences for the 12 participants were as follows: BADCE, ACBDE, CBAED, ABCDE, DCBAE, ABCED, DCBAE, ABCED, EABDC, CDBAE, ABCED, and DCBAE (where A = 3.2 nmol/kg of kisspeptin-54, B = 6.4 nmol/kg, C = 12.8 nmol/kg, D = 25.6 nmol/kg, and E = placebo). For the study in healthy women and patients with HA, the intervention sequences were either AB or BA (where A = 12.8 nmol/kg of kisspeptin-54, and B = placebo). The assignment schedule was held centrally at Imperial College London (password protected) and concealed from the study team (accessible only to the principal authors EGM, ANC, and WSD). Study participants, study visit doctors, and research nurses interacting with the participants, and reproductive hormone analysts were blinded to the identity of the interventions.

#### Outcome measures

The primary outcome measure was change in serum LH following intranasal administration of kisspeptin compared to placebo. The secondary outcome measures were changes in serum FSH, as well as testosterone [in men], and oestradiol and progesterone [in women] following intranasal administration of kisspeptin compared to placebo. In addition, safety assessments were performed that included adverse event recording, blood pressure, and heart rate measurements.

#### Sample size

This was the first study to examine the effects of intranasal kisspeptin administration on reproductive hormone secretion in humans. However, our previous work in healthy volunteers demonstrates that *intravenous* kisspeptin increases serum LH by mean 10.2 IU/L and standard deviation 0.4 IU/L, compared with placebo administration (mean 2.0 IU/L, standard deviation 0.1 IU/L),^54^ and we anticipated a similar response following intranasal administration. Using these data, with α = 0.05, power = 0.8 and effect size = 0.997, a power calculation was performed resulting in a final sample size required of 10 participants per study group (i.e., healthy men, healthy women, and patients with HA). Although this sample size calculation was based on previous data obtained in healthy volunteers,^54^ it is well-established that the gonadotropin response following kisspeptin administration is heightened in patients with HA^4^ and so a sample size of 10 was deemed sufficient to detect a significant effect in the patient group. Moreover, a sample size of 10 per study group is regarded typical of proof-of-concept studies and is considered adequate for the objectives of the study to be achieved.^55^ In addition, our sample size of 10 per study group compares favourably with our previous work examining the effects of intravenous and subcutaneous kisspeptin administration on reproductive hormone secretion in both healthy volunteers and patients with reproductive disorders.^4^^,^^54^^,^^56^, ^57^, ^58^ No interim analysis of the dataset was undertaken to guide the sample size. Recruitment was stopped when sufficient numbers had enrolled to ensure the planned sample size was achieved, and the study was stopped once all participants had completed the study protocol, as no safety concerns occurred requiring early termination.

#### Intranasal administration

Kisspeptin-54 or placebo was self-administered using nasal spray devices that delivered a single volume of 100 μL per actuation (SP270+ pump, 3959 actuator, 5 mL SGD U-Save glass bottles provided by Nemera, La Verpillière, France). The kisspeptin-54 nasal spray solution formulations were prepared by reconstituting kisspeptin-54 powder stored in sterile vials on the morning of each study visit to produce a solution containing the target dose according to body weight in 400 μL.

Participants were familiarised with the procedure of intranasal application and were provided with written administration instructions ahead of each study visit, as described in.^38^ In addition, participants received a 5-min training session using a dummy nasal spray device before starting the study to ensure good technique, which all participants easily understood. Under instruction and supervision from the clinical team, participants primed the nasal sprays then self-administered a total of four sprays of kisspeptin-54 or placebo per visit (i.e., 4 × 100 μL), alternating between nostrils and leaving 60 s between sprays, to allow satisfactory dissipation and avoid solution running out of the nostrils. Nasal sprays containing kisspeptin or placebo were identical in appearance, volume, and administration technique.

#### Kisspeptin-54 peptide

Kisspeptin-54 was synthesised by Bachem (Bachem Holding AG) and purified by reverse-phase high-performance liquid chromatography (HPLC). Sterile vials of kisspeptin-54 were produced by Bachem (Clinalfa, Bachem Distribution Services GmbH) according to Good Manufacturing Practice. Electrospray mass spectroscopy and amino acid analysis confirmed the identity of the peptide as previously described.^54^ The *Limulus* amoebocyte lysate assay (Associates of Cape Cod, Liverpool, UK, catalogue #T0051-25) was negative for endotoxin, and the peptide was sterile on culture (Department of Microbiology, Hammersmith Hospital, London). Vials of freeze-dried kisspeptin-54 were stored at −20 °C.

The kisspeptin doses selected were based on our previous data demonstrating robust LH rises following a single intravenous bolus of 6.4, 12.8, and 25.6 nmol/kg of kisspeptin-54 in healthy men,^59^ as well as the addition of a lower dose of 3.2 nmol/kg given that administration of kisspeptin has until now not been investigated via the intranasal route in humans.

#### Hormone assays

Blood samples were collected to measure serum reproductive hormone levels at the timepoints depicted in Fig. 1, Fig. 2, Fig. 3a. Serum samples were stored at −20 °C until analysis. Serum LH, FSH, testosterone (in men), and oestradiol and progesterone (in women) were measured using automated chemiluminescent immunoassays (Abbott Diagnostics, Maidenhead, UK: LH catalogue # 2P40-25, FSH catalogue # 7K75-25, testosterone catalogue # 07K7320, oestradiol catalogue # 7K72-25, progesterone catalogue # 7K77-25). Reference ranges: LH in international units per litre (IU/L), 2–12 (men), 2–10 (follicular), 20–60 (mid-cycle), 4–14 (luteal); FSH in IU/L, 1.7–8 (men), 1.5–8.0 (follicular and luteal), 10–50 (mid-cycle); in men: testosterone in nanomoles per litre (nmol/L), 10–30; and in women: oestradiol in picomoles per litre (pmol/L), <300 (early follicular), 400–1500 (mid-cycle), 200–1000 (luteal) and progesterone in nmol/L, <5.0 (follicular), 3.5–67 (luteal). Intra-assay and inter-assay coefficients of variation: LH, 2.7 and 4.1%; FSH, 3.0 and 4.1%; total testosterone, 2.8 and 4.2%; oestradiol, 3.0 and 3.4%; progesterone 2.4 and 2.9%. Limits of detection: LH, 0.07 IU/L; FSH, 0.05 IU/L; total testosterone, 0.05 nmol/L; oestradiol, 70 pmol/L; progesterone, 0.3 nmol/L. AMH was measured using an enzyme linked immunosorbent assay (Beckman Coulter Inc, Brea, CA, USA, catalogue #B13127). The reference range was 2.2–48.5 pmol/L, lower limit of detection 0.6 pmoL/l and upper limit of detection 68.9 pmol/L.

#### Statistics

Statistical analyses were performed using GraphPad Prism (version 9.4.1). Data are presented as mean ± SEM. Normality of the data was determined using the Shapiro–Wilk test. Time profiles of hormone levels during the 4-h blood sampling were compared using two-way ANOVA with Bonferroni multiple comparison test. Means were compared using one-way ANOVA with Bonferroni multiple comparison test (men), paired t-tests (healthy women and women with HA), and unpaired t-tests (healthy women vs. women with HA). In all cases, *P* < 0.05 was considered statistically significant. All data of serum reproductive hormones are presented as the change from baseline (i.e., average of timepoints −30, −15, and 0 min) in serum levels after intranasal administration of kisspeptin-54 or placebo.

### Pharmaceutical studies of intranasal kisspeptin-54

#### Deposition of Kisspeptin-54 sprays in a nasal cast

The deposition pattern of the nasal spray in the nasal cavity was characterised *in vitro* using an Alberta Idealised Nasal Inlet (AINI; Copley Scientific, UK), which has sections representing the nasal vestibule, turbinates, olfactory region, and nasopharynx.^60^ Nasal sprays containing kisspeptin-54 (3.5 mg/mL) were administered to the cast operated with an inspiration air flow of 7.5 L/min. The nasal spray was inserted into the nostril of the cast at an angle and penetration depth to mimic the administration procedure in the human clinical study. Two sprays were administered for each determination at an angle of 45° parallel to the septum. The cast was dissembled immediately after administration of the sprays to minimise redistribution of the deposited dose. Kisspeptin-54 was recovered from individual cast segments by rinsing with 2 mL saline and quantified using HPLC with all measurements performed in triplicate.

#### Stability of kisspeptin-54 solutions for nasal delivery

Kisspeptin-54 3.5 mg/mL solutions, equivalent to the lowest dose used in the human clinical study, were prepared and aliquoted into glass nasal spray devices and sealed amber glass vials (airtight, light protected control). The test materials were stored upright in a single location (4 °C refrigerator with temperature monitoring) with minimal manipulation or movement for 60 days. On each day of analysis, three aliquots were sampled from each container and analysed by HPLC.

#### HPLC analysis

Each kisspeptin-54 sample was assayed in triplicate using a Waters 2795 Separation Module HPLC equipped with Waters 2996 Photodiode Array Detector and a Luna® 3 μm C18 100 Å LC Column 150 × 4.6 mm with trimethylsilyl (TMS) end capping used. Injection volume was 25 μL and the mobile phase was a gradient using eluent A (0.05% trifluoroacetic acid (TFA):1% acetonitrile (ACN): 98.95% H_2_0) and eluent B (0.05% TFA in MeOH/ACN 1:1(V/V)) from 20 to 60% eluent B with a flow rate of 1 mL/min and a run time of 15 min. Column temperature was set at 40 °C and ultraviolet (UV) detection at 220 nm. The assay was validated for linearity (r = 0.993), accuracy and precision in the range 0.3–0.7 mg/mL. Assay specificity was confirmed by using conditions to force kisspeptin-54 degradation (2 M HCL or phosphate buffer at 60 °C for 72 h) resulting in loss or reduction of the kisspeptin peak and appearance of non-interfering additional peaks.

#### Kisspeptin-54 powder particle size and shape analysis

Particle size and shape analysis of the kisspeptin-54 powder was performed using a Morphologi 4 (Malvern Instruments, Worcestershire, UK). Particles were readily dispersed using the dispersion unit at low energy level. Bright field illumination was used for microscopic visualisation of the particles with the light intensity calibrated to 70%. The samples were scanned using the 20× objective lens over a scan area of 4.5 mm by 4.5 mm with a trash size of 10 pixels.

### Effects of intranasal administration of kisspeptin in mice

#### Ethics

The animal studies were approved by the Institutional Ethics Committees for the Care and Use of Experimental Animals of the University of Lille (France) and the French Ministry of National Education, Higher Education and Research (APAFIS number 2617- 2015110517317420 v5 and APAFIS number 13387-2017122712209790 v9). All experiments were performed in accordance with the guidelines for animal use specified by the European Union Council Directive of 22nd September 2010 (2010/63/EU) and were conducted in accordance with the ARRIVE (Animal Research: Reporting of *in vivo* Experiments) guidelines. All efforts were made to minimise animal suffering and animal care was supervised by veterinarians and animal technicians skilled in rodent healthcare and housing.

#### Animals

Adult C57BL/6J male mice were purchased from Charles River Laboratories (USA) and were housed under specific pathogen-free conditions in a temperature-controlled (21–22 °C) and humidity-controlled (30–60%) environment, with a 12-h light–dark cycle and ad libitum access to food and water. They were housed in individually ventilated cages, with a maximum of six mice per cage. A standard diet (9.5 mm Pelleted RM3, Special Diets Services; Competence Centre for Lab Animal Science of SAFE®; France) was given to all mice during the experimental period. All animals were naïve, and all experimental procedures commenced when mice were 8–10 weeks old and ended when mice were 12–14 weeks old. As frequent in non-human studies, precise power calculations were not used to predetermine sample sizes in each part of the rodent studies, but our sample sizes compare favourably to those reported in previous publications^61^^,^^62^ and were approved by the local ethics committee (University of Lille, France). Animals were randomised to experimental groups and investigators were blinded to group allocation during data collection and analyses to minimise any potential bias. No samples or animals were excluded from the analysis.

#### Effects of intranasal kisspeptin-54 administration on plasma LH levels in male mice

Saline (placebo) or kisspeptin-54 were intranasally administered using a customised and unsharpened 2 mm long and 0.3 mm wide hypodermic needle (BD PrecisionGlide™; 30G; ref. 305106). Male mice were restrained and a single volume of 30 μL of the solution was delivered in one randomly chosen nostril with either 0.9% saline or different concentrations of kisspeptin-54 (1, 3, 12.8, 30, or 50 nM; equivalent to 0.001–0.05 nmol/kg). The protocol involved *N* = 5 mice per group, except concentrations 12.8 nM where *N* = 6 and 30 nM where *N* = 7. The kisspeptin concentrations selected were based on previous data that 1 nM of kisspeptin-54 robustly increases LH when administered by the intraperitoneal route^63^; this concentration (1 nM) and higher concentrations (3, 12.8, 30, or 50 nM) were therefore selected. LH levels were assessed using a tail-tip serial blood sampling as previously reported.^64^^,^^65^ Briefly, samples containing 4 μL of whole blood were collected every 10 min over a 70-min-long period from each animal. The first sampling point was taken as baseline LH level (−5 min), and saline or kisspeptin-54 were administered at timepoint 0 min. Whole blood was collected into Eppendorf tubes pre-loaded with 50 μL of 0.1 M phosphate-buffered saline (PBS)-0.05% Tween, thoroughly mixed, and snap-frozen with dry ice and stored at −80 °C.

#### Mouse LH measurements and analysis

LH levels were measured using an established in-house and ultrasensitive mouse LH ELISA method as previously validated.^66^ A primary coating antibody (bovine LHβ subunit, 518B7; L. Sibley; University of California, UC Davis, RRID AB_2756886) was used at a 1:1000 dilution in combination with a detection primary antibody (rabbit LH antiserum, AFP240580Rb, RRID AB_2665533; NIDDK- NHPP) used at 1:10,000 dilution, and a secondary horseradish peroxidase-conjugated antibody (goat anti-rabbit; Vector Laboratories, PI-1000, RRID AB_2336198) used at 1:10,000 dilution. The assay standard curve used a mouse LH-RP (AFP5306A, A. F. Parlow, National Hormone and Peptide Program; supplied by Golden West BioSolutions, California, USA; catalogue # TLIA1053.03). The assay sensitivity of this LH ELISA was 0.04 ng/mL, intra-assay coefficient of variation 4.1%, and inter-assay coefficient of variation 7.3%.

#### Statistics

Statistical analyses were performed using GraphPad Prism (version 9.4.1). Data are presented as mean ± SEM. Normality of the data was determined using the Shapiro–Wilk test. The analysis considered increment in LH levels when average post-administration values were above significance (*P* < 0.05) compared to basal levels (−5 min) using repeated measures two-way ANOVA with Fishers least significant difference (LSD) test. Integrated LH response to kisspeptin-54 administration as indicated using the area under the curve (AUC) analysis when all peaks were above baseline values. The difference in AUC between groups was compared by one-way ANOVA with Fisher’s LSD test. In all cases, *P* < 0.05 was considered statistically significant.

#### Fluorescently tagged Kisspeptin-54

Kisspeptin-54 labelled with a D2 fluorescent probe (kisspeptin-54–D2) was developed by (Cisbio Bioassays, France). The D2 dye was incorporated in the N-terminal region by adding a cysteine residue before the last glycine of kisspeptin-54 (Dye-C-GTSLSPPPESSGSRQQPGLSAPHSRQIPAPQGAVLVQREKDLPNYNWNSFGLRF). The compound was resuspended in dimethyl sulfoxide (DMSO), and further diluted in saline before administration.

#### Fluorescence *in situ* hybridisation (FISH)

Fresh-frozen 14 μm coronal sections containing the OB of male mice (Bregma +3.4–3.6 mm) were prepared using a cryostat (CM3050S, Leica). FISH was performed with the RNAscope Multiplex Fluorescent Kit v2 according to the manufacturer’s protocol (Advanced Cell Diagnostics, ACD, catalogue # 323110). Specific probes were used to detect *Gnrh1* and *Kiss1r* mRNAs:

• Mm-GnRH-O1-C3–ref:476281-C3; provider: ACD.
• Mm-Kiss1r—ref:408001; provider: ACD.

#### Image analysis

Confocal observations and analyses were performed with an inverted laser scanning Axio Observer microscope (LSM710, Zeiss, Oberkochen, Germany) equipped with EC Plan NeoFluar 20×/0.5 NA, 40×/1.3 NA and 63×/1.8 NA (Zeiss) objectives (Imaging Core Facility of the University of Lille, France). ImageJ (version 2, National Institute of Health, Bethesda, USA) and Photoshop (version 24.0.1, Adobe Systems, San Jose, USA) were used to process and quantify images and to prepare the figures.

#### Intranasal administration of kisspeptin-54-d2, tissue clearing and 3D imaging

C57BL/6J wild-type male mice were anesthetised with 2% isoflurane before intranasal administration of a fluorescently tagged kisspeptine-54 (kisspeptin-54-D2; 12.8 nM in 30 μL NaCl 0.9%). Mice were deeply anesthetised 25 min after intranasal administration of kisspeptin-54-D2 by intraperitoneal injection of pentobarbital (200 mg/kg) and transcardially perfused with a saline solution (0.9% NaCl) followed by a fixative solution (4% paraformaldehyde in PBS 0.01 M, pH 7.4). Whole heads were post-fixed overnight at 4 °C and rinsed in PBS (0.01 M, pH7.4).

Following fixation, the heads of injected animals were decalcified for 2 × 24 h at RT in a large amount of decalcification solution (20% EDTA in ddH2O, adjusted to pH 8). After extensive rinses in PBS 0.01 M to wash out the decalcification solution, the unnecessary tissues were carefully removed from the heads to facilitate imaging. Tissue clearing was performed as described in the immunolabeling-enabled imaging and automated cell detection of solvent-cleared organs (iDISCO+) protocol.^67^ Notably, this protocol has been previously described in other publications,^68^, ^69^, ^70^ showing that the immunolabeling is not compromised by the prolonged decalcification steps. In brief, sample dehydration was carried out in a methanol/PBS gradient (20%, 40%, 60%, 80%, 100%, 100%, 1 h each) and immediately followed by an overnight delipidation in 66% dichloromethane/33% methanol. On the following day, the methanol was washed out in 100% dichloromethane for 1 h, before the samples were optically cleared by a 3-h incubation in dibenzyl ether.

Cleared heads were imaged in sagittal orientation on the Ultramicroscope I equipped with a 1.1×/0.1NA MI PLAN immersion objective (Miltenyi Biotec). The acquisition coordinates were set to cover a 3.5 mm-thick region encompassing the medial part of the head and ranging from the nose to the cerebellum. The numerical aperture of the lightsheet was set to 0.03 and a z-step of 4 μm was defined. The green (488, tissue autofluorescence) and far red (647, kisspeptin-54-D2) channels were acquired and saved as a sequence of OME-TIFF files. Imaris 9.6 (Bitplane) was used for conversion, visualisation, and analysis of the lightsheet datasets.

#### Chemicals

• Dibenzyl ether—ref: 148400010; provider: Thermo Scientific.
• Dichloromethane—ref: 270997; provider: Sigma.
• EDTA disodium salt—ref: E5134; provider: Sigma.
• Methanol—ref: 20847.240; provider: VWR International.

### Role of funders

The funding sources played no role in the study design and protocol, data collection, monitoring, analysis, interpretation, writing or editing of the manuscript.

## Results

### Effects of intranasal administration of kisspeptin in healthy men

It is not known whether *intranasal* administration of kisspeptin stimulates reproductive hormone release. As such, we first conducted clinical studies in twelve healthy men (mean age 28.3 ± 1.7 years, BMI 24.5 ± 0.7 kg/m^2^) as part of a randomised, double-blinded, crossover, placebo-controlled study (Fig. 1a and Supplemental Fig. S1). A summary of baseline characteristics is provided in Table 1. Importantly, reproductive hormone levels were equivalent between study visits at baseline (Supplemental Table S1). Participants attended for five study visits each (with visits separated by one week) and received all the following five interventions, in random order, via the intranasal route: 3.2, 6.4, 12.8, and 25.6 nmol/kg of kisspeptin-54, or 0.9% saline (placebo). These doses were selected based on previous studies in human volunteers where subcutaneous administration of these doses of kisspeptin-54 resulted in a significant LH rise.^16^^,^^59^ After brief instructions, all participants self-administered intranasal kisspeptin-54 or placebo, followed by blood sampling for serum LH, FSH, and testosterone every 15 min for 4 h.

**Table 1:** Baseline characteristics of healthy participants and patients with hypothalamic amenorrhoea.

	Healthy Men (*N* = 12)	Healthy Women (*N* = 12)	Women with HA (*N* = 10)	*P*-value
Age (years)	28.3 ± 1.7	22.1 ± 0.9	25.8 ± 2.7	0.101
Weight (kg)	75.4 ± 1.9	61.1 ± 3.1	57.1 ± 3.4	0.400
BMI (kg/m^2^)	24.5 ± 0.7	22.1 ± 0.8	19.9 ± 1.3	0.107
Ethnic group				
Asian	5	3	2	–
Black	0	0	0	–
Mixed	0	3	1	–
White	7	6	7	–
Other	0	0	0	–
Baseline reproductive hormones				
LH (IU/L)	3.5 ± 0.3	4.3 ± 0.7	1.4 ± 0.8	**<0.001∗∗**^a^
FSH (IU/L)	4.0 ± 1.0	5.0 ± 0.6	4.0 ± 0.5	0.341
Testosterone (nmol/L)	17.7 ± 1.4	–	–	–
Oestradiol (pmol/L)	–	324.3 ± 61.5	102.1 ± 0.0	**0.004∗∗**^b^
SHBG (nmol/L)	27.3 ± 4.1	55.0 ± 9.9	78.0 ± 21.0	0.142
AMH (pmol/L)	–	19.1 ± 2.9	35.1 ± 9.7	**0.017∗**^c^

Mean ± SEM are presented. AMH, anti-Müllerian hormone; BMI, body mass index; FSH, follicle-stimulating hormone; HA, hypothalamic amenorrhoea; LH, luteinising hormone; SHBG, sex hormone binding globulin.

*P*-values represent comparisons between healthy women and women with HA by unpaired t-tests.

a Mean difference = 3.0 IU/L [95% CI, 1.5–4.4], *P* < 0.001.

b Mean difference = 222.2 pmol/L [95% CI, 81.0–363.3], *P* = 0.004.

c Mean difference = 16.0 pmol/L [95% CI, 3.2–28.8], *P* = 0.017.

#### Intranasal kisspeptin acutely stimulates gonadotropin release in healthy men

Intranasal kisspeptin-54 administration to healthy men resulted in rapid clinically-significant and dose-dependent increases in circulating LH levels, followed by sustained elevations throughout the sampling period, in comparison to placebo (6.4 nmol/kg vs. placebo mean difference = 1.3 IU/L [95% CI, 0.4–2.2], *P* = 0.006, two-way ANOVA with Bonferroni correction; 12.8 nmol/kg vs. placebo mean difference = 2.5 IU/L [95% CI, 1.5–3.5], *P* < 0.001, two-way ANOVA with Bonferroni correction; 25.6 nmol/kg vs. placebo mean difference = 2.3 IU/L [95% CI, 1.2–3.4], *P* < 0.001, two-way ANOVA with Bonferroni correction). Peak stimulation of serum LH was observed 30–45 min after intranasal kisspeptin-54 administration (Fig. 1b). Correspondingly, the mean AUC increase in serum LH was elevated significantly compared to placebo, following all doses of kisspeptin-54: mean AUC increase (in h·IU/litre) 3.2 nmol/kg: 172.2 ± 64.2 (mean difference = 197.6 [95% CI, 10.6–384.6], *P* = 0.037, one-way ANOVA with Bonferroni correction); 6.4 nmol/kg: 300.2 ± 79.2 (mean difference = 325.5 [95% CI, 122.6–528.4], *P* = 0.002, one-way ANOVA with Bonferroni correction); 12.8 nmol/kg: 595.7 ± 98.3 (mean difference = 621.1 [95% CI, 257.2–985.1], *P* = 0.001, one-way ANOVA with Bonferroni correction); 25.6 nmol/kg: 549.0 ± 108.6 (mean difference = 574.4 [95% CI, 353.7–795.1], *P* < 0.001, one-way ANOVA with Bonferroni correction) (Fig. 1c and Supplemental Table S2). Similarly, the mean maximal increase in LH from baseline occurred in a dose-dependent manner, with maximal stimulation of LH observed after kisspeptin-54 at 12.8 nmol/kg: 4.4 ± 0.6 IU/L above baseline (mean difference = 3.1 IU/L [95% CI, 1.2–4.9], *P* = 0.002 vs. placebo, one-way ANOVA with Bonferroni correction) (Fig. 1d and Supplemental Table S2).

Similar to LH, serum FSH levels were significantly elevated compared to placebo following intranasal kisspeptin-54 administration at doses 6.4 nmol/kg (mean difference = 0.4 IU/L [95% CI, 0.1–0.7], *P* = 0.005 vs. placebo, two-way ANOVA with Bonferroni correction) and 12.8 nmol/kg (mean difference = 0.6 IU/L [95% CI, 0.3–0.9], *P* < 0.001 vs. placebo, two-way ANOVA with Bonferroni correction) (Fig. 1e). Maximal overall stimulation of FSH was observed after 12.8 nmol/kg, with mean AUC increase 96.3 ± 23.2 h·IU/litre (mean difference = 156.6 [95% CI, 14.2–299.0], *P* = 0.029 vs. placebo, one-way ANOVA with Bonferroni correction) (Fig. 1f and Supplemental Table S2) and peak FSH rise of 0.7 ± 0.2 IU/L above baseline (mean difference = 0.7 IU/L [95% CI, 0.1–1.3], *P* = 0.022 vs. placebo, one-way ANOVA with Bonferroni correction) (Fig. 1g and Supplemental Table S2).

#### Intranasal kisspeptin stimulates testosterone release in healthy men

Following intranasal kisspeptin-54 administration (12.8 nmol/kg), there was an increase in downstream testosterone (Fig. 1h). At this dose, serum levels of testosterone steadily increased to peak levels from 120 min (mean difference = 1.91 nmol/L [95% CI, 0.26–3.55], *P* = 0.03, two-way ANOVA with Bonferroni correction) after administration, with this rise delayed compared to LH and FSH as expected for a downstream hormone. Following 12.8 nmol/kg, the mean AUC increase was 470.4 ± 144.0 h nmol/L (mean difference = 654.3 [95% CI, 224.6–1084.0], *P* = 0.003 vs. placebo, one-way ANOVA with Bonferroni correction) (Fig. 1i and Supplemental Table S2), with a clinically-significant maximal increase of 4.9 ± 0.7 nmol/L above baseline at 150 min (mean difference = 2.4 nmol/L [95% CI, 0.4–4.4], *P* = 0.018 vs. placebo, one-way ANOVA with Bonferroni correction) (Fig. 1j and Supplemental Table S2).

### Effects of intranasal administration of kisspeptin in healthy women

Having shown that intranasal administration of kisspeptin-54 robustly and dose-dependently stimulates gonadotropin release in healthy men, we next sought to determine whether this could be recapitulated in a group of 12 healthy (ovulatory) women (mean age 22.1 ± 0.9 years, BMI 22.1 ± 0.8 kg/m^2^). A summary of baseline characteristics is provided in Table 1. Healthy women completed two visits each, conducted in the follicular phase of the menstrual cycle (i.e., days 2–10 inclusive), and were administered the following two interventions in random order: intranasal kisspeptin-54 at 12.8 nmol/kg and placebo (with one intervention administered per menstrual cycle) (Fig. 2a and Supplemental Fig. S2). This 12.8 nmol/kg dose of intranasal kisspeptin-54 was chosen as in the healthy men study (above) this dose was observed to have maximal effect on gonadotropin release. Importantly, reproductive hormone levels were equivalent between study visits at baseline (Supplemental Table S3).

#### Intranasal kisspeptin acutely stimulates gonadotropin release in healthy women

Similar to our observations in healthy men, intranasal kisspeptin-54 administration to healthy women induced a rapid increase in serum LH levels over time, compared to placebo (mean difference = 0.7 IU/L [95% CI, 0.3–1.2], *P* = 0.002 vs. placebo, two-way ANOVA with Bonferroni correction) (Fig. 2b). Following intranasal kisspeptin-54, LH release was elevated significantly compared to placebo: mean AUC increase 96.0 ± 45.8 h·IU/litre, compared with −83.8 ± 22.3 h·IU/litre following placebo (mean difference = 179.8 [95% CI, 89.2–270.5], *P* = 0.001, paired t-test) (Fig. 2c and Supplemental Table S4). Furthermore, the maximal increase in serum LH following 12.8 nmol/kg was 1.4 ± 0.3 IU/L above baseline, compared with 0.4 ± 0.1 IU/L following placebo (mean difference = 1.0 IU/L [95% CI, 0.4–1.7], *P* = 0.004, paired t-test) (Fig. 2d and Supplemental Table S4).

Unlike the robust effects on LH, the effects of intranasal kisspeptin administration on FSH release were less pronounced in healthy women. Following intranasal kisspeptin-54, serum FSH levels were higher over time compared to placebo, albeit below baseline and non-significantly (mean difference = 0.3 IU/L [95% CI, 0.0–0.7], *P* = 0.073 vs. placebo, two-way ANOVA with Bonferroni correction) (Fig. 2e). Similarly, FSH release was significantly higher compared to placebo, albeit below baseline: mean AUC increase −36.1 ± 23.4 h·IU/litre, compared with −112.5 ± 33.1 h·IU/litre following placebo (mean difference = 76.4 [95% CI, 13.4–139.4], *P* = 0.022, paired t-test) (Fig. 2f and Supplemental Table S4). Moreover, the maximal increase in serum FSH following 12.8 nmol/kg was 0.3 ± 0.1 IU/L above baseline, compared with −0.1 ± 0.1 IU/L following placebo (mean difference = 0.4 IU/L [95% CI, 0.1–0.7], *P* = 0.019, paired t-test) (Fig. 2g and Supplemental Table S4).

#### Effects of intranasal kisspeptin on serum oestradiol and progesterone in healthy women

Intranasal kisspeptin-54 administration had no significant effects on downstream circulating oestradiol and progesterone levels during this acute 4-h time-course (Supplemental Fig. S3a and b).

### Effects of intranasal kisspeptin in patients with reproductive disorders

We next investigated if the observed stimulatory effects on gonadotropin release in healthy men and women could be translated into a patient group of 10 anovulatory women with one of the commonest reproductive disorders, HA, (mean age 25.8 ± 2.7 years, BMI 19.9 ± 1.3 kg/m^2^) as this could provide a novel therapeutic option, preferable to clinicians and patients. A summary of baseline characteristics is provided in Table 1. Patients with HA were comparable in age to the healthy women (above), whereas they had a trend to lower weight and BMI, as expected. Furthermore, the patients with HA differed in terms of lower baseline LH and oestradiol levels, and higher AMH levels, compared to the healthy women, consistent with the typical biochemical picture observed in HA.^50^, ^51^, ^52^ Patients completed two visits each, separated by at least one week, and were administered the following two interventions in random order: intranasal kisspeptin-54 at 12.8 nmol/kg and placebo (Fig. 3a and Supplemental Fig. S4). Importantly, reproductive hormone levels were equivalent between study visits at baseline (Supplemental Table S5).

#### Intranasal kisspeptin acutely stimulates gonadotropin release in patients with HA

Intranasal kisspeptin-54 administration to patients with HA induced an acute and potent increase in serum LH levels over time, compared to placebo (mean difference = 3.0 IU/L [95% CI, 1.7–4.4], *P* < 0.001 vs. placebo, two-way ANOVA with Bonferroni correction) with maximal stimulation achieved from 30 to 45 min post-administration (Fig. 3b). In response to intranasal kisspeptin-54, LH release was elevated significantly compared to placebo: mean AUC increase 600.6 ± 146.7 h·IU/litre, compared with −156.3 ± 49.0 h·IU/litre following placebo (mean difference = 756.9 [95% CI, 444.9–1069.0], *P* < 0.001, paired t-test) (Fig. 3c and Supplemental Table S6). Moreover, the maximal increase in serum LH following 12.8 nmol/kg was a clinically-significant 4.4 ± 0.2 IU/L above baseline, compared with 0.1 ± 0.2 IU/L following placebo (mean difference = 4.3 IU/L [95% CI, 2.7–6.0], *P* < 0.001, paired t-test) (Fig. 3d and Supplemental Table S6). Notably, this was over 3-fold higher than what we observed with the same kisspeptin-54 dosing regimen in healthy women (mean difference = 2.95 IU/L [95% CI, 1.48–4.41], *P* < 0.001, unpaired t-test) (Fig. 3h).

In response to intranasal kisspeptin-54, serum FSH levels increased over time and remained elevated throughout the study duration, compared to placebo (mean difference = 2.35 IU/L [95% CI, 0.31–4.40], *P* = 0.026 vs. placebo, two-way ANOVA with Bonferroni correction) (Fig. 3e). Furthermore, FSH release was elevated significantly compared to placebo: mean AUC increase 474.9 ± 237.3 h·IU/litre, compared with −104.7 ± 26.5 h·IU/litre following placebo (mean difference = 580.0 [95% CI, 38.5–1121.0], *P* = 0.038, paired t-test) (Fig. 3f and Supplemental Table S6). The maximal increase in serum FSH following 12.8 nmol/kg was 3.1 ± 0.3 IU/L above baseline, compared with 0.0 ± 0.1 IU/L following placebo (mean difference = 3.1 IU/L [95% CI, 0.1–6.1], *P* = 0.049, paired t-test) (Fig. 3g and Supplemental Table S6). Again, this was over 10-fold higher than what we observed with the corresponding 12.8 nmol/kg dose of kisspeptin-54 in healthy women (mean difference = 2.77 IU/L [95% CI, 0.26–5.28], *P* = 0.032, unpaired t-test) (Fig. 3I).

#### Effects of intranasal kisspeptin on serum oestradiol and progesterone in patients with HA

Intranasal kisspeptin-54 administration had no significant effects on downstream circulating oestradiol and progesterone levels during this acute 4-h time-course and similar to the healthy women group (Supplemental Fig. S5a and b).

#### Safety assessments in healthy participants and patients

Prior to this study, kisspeptin had been administered using the *intravenous* and *subcutaneous* routes to over 1000 healthy men and women, as well as patients with reproductive and psychosexual disorders, without any observed adverse effects^71^ and therefore no safety concerns were anticipated with *intranasal* kisspeptin administration. In keeping with this and importantly from a pharmaceutical development perspective, intranasal kisspeptin administration was well-tolerated by all participants with no side effects or adverse events (of any severity) reported by any participant in any of the study groups (Supplemental Tables S7–S9). Furthermore, from a medicines safety perspective, intranasal administration of kisspeptin-54 had no effect on blood pressure or heart rate measurements at any of the doses administered.

### Pharmaceutical studies of intranasal kisspeptin

Our data demonstrate that intranasal kisspeptin-54 administration significantly stimulates gonadotropin release in healthy men and women, as well as patients with HA. However, to be a clinical therapeutic in patients with reproductive disorders, clinical translation of intranasal kisspeptin-54 depends critically on the performance of the intranasal nasal spray device employed and the chemical stability of kisspeptin-54 in solution for nasal delivery. Therefore, we next undertook a series of pharmaceutical studies to evaluate the deposition profile of the nasal kisspeptin-54 spray in the nasal cavity and the chemical stability of kisspeptin-54 in real-time during storage at refrigerator temperature.

#### Nasal deposition of kisspeptin sprays

**Fig. 4:**
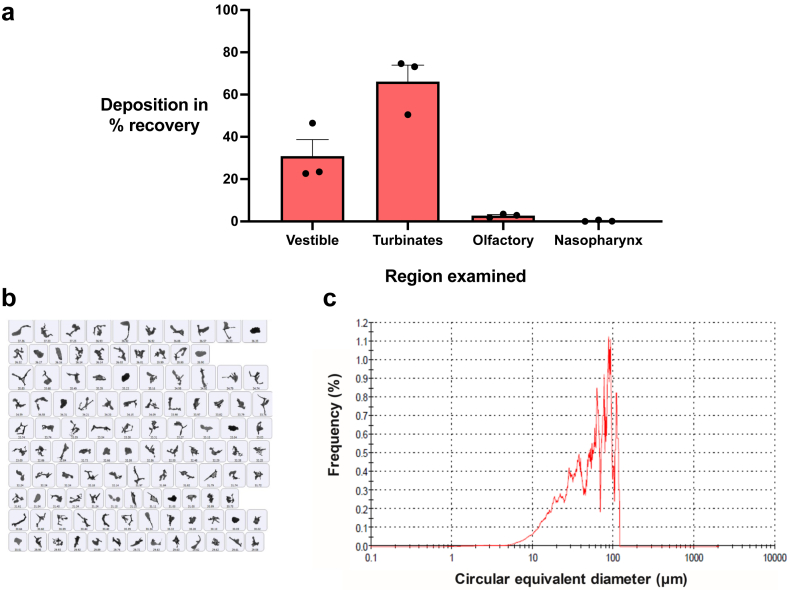
**Pharmaceutical studies. (a)** Deposition profile of kisspeptin-54 in 0.9% saline solution nasal spray. Proportion of recovered dose depositing in the nasal vestibule, turbinates, olfactory region, and nasopharynx sections of the Alberta Idealised Nasal Inlet, an idealised replica of the adult nasal cavity. Recovery was 96.5% of the emitted dose; data represent *N* = 3 with SEM. **(b and c)** Size and shape of kisspeptin-54 powder particles measured using the Morphologi 4. **(b)** Shape of representative kisspeptin-54 powder particles in the 20–50-micron circular equivalent diameter size range. **(c)** Particle size distribution of kisspeptin-54 powder particles (*N* = 55,421), showing 0.5–113 μm circular equivalent diameter range and over 40% of the particles in the optimal 20–50 μm range for nasal delivery.

We characterised the regional deposition pattern of the nasal spray in the nasal cavity *in vitro* using an idealised replica of the human adult nasal cavity, which has sections representing the nasal vestibule, turbinates, olfactory region, and nasopharynx.^60^ Following administration of nasal sprays containing kisspeptin-54 (3.5 mg/mL), the cast was dissembled, and kisspeptin-54 was recovered from individual cast segments and quantified using HPLC. Using this technique, regional deposition was reproducible (Fig. 4a) with no visible liquid pooling or signs of post-deposition redistribution. Recovery of drug from the nasal cavity was 96.5 ± 5.2% of the emitted dose. Approximately 70% of the spray was delivered beyond the nasal vestibule with the majority depositing in the turbinates (66.1 ± 13.5%) and a smaller proportion depositing in the olfactory region (2.7 ± 0.9%). The amount reaching the nasopharynx was minimal (0.2 ± 0.4%). This represents ideal properties of an intranasally administered drug, whereby it is important to have predictable deposition and preferably within the turbinate region, an area rich in olfactory neuroepithelium.^72^

#### Stability of kisspeptin solutions for nasal delivery

The chemical stability of kisspeptin-54 in 0.9% saline solution when stored in glass nasal spray devices and sealed amber glass vials (airtight, light protected control) at 4 °C was determined by measuring the concentration of kisspeptin-54 by HPLC at five timepoints over 60 days. Kisspeptin-54 was stable in solution when stored in sealed amber glass vials, with the concentration remaining within the acceptable threshold for pharmaceutical stability, i.e., 5% reduction of the initial concentration^73^ (Table 2). After 60 days, the concentration of kisspeptin-54 was reduced by 5.4% and 5.0% of the initial concentration in the nasal spray devices and sealed amber glass vials, respectively. The kisspeptin-54 solution appeared as a clear solution when freshly made and no change was observed over 60 days of storage in either container type.

**Table 2:** Stability of kisspeptin-54 in 0.9% saline solution for nasal delivery.

Storage conditions	% of initial concentration (mean ± SEM, *N* = 3)				
	4 days	7 days	14 days	28 days	60 days
4 °C in sealed amber vial	98.94 ± 0.57	98.69 ± 0.58	97.91 ± 0.64	97.32 ± 0.56	95.01 ± 1.45
4 °C in nasal spray	98.06 ± 1.11	98.28 ± 0.69	97.90 ± 0.67	96.11 ± 0.51	94.56 ± 1.67

Stability of kisspeptin-54 (3.5 mg/mL) over 4, 7, 14, 28 and 60 days of storage. Mean ± SEM are presented. *N* = 3.

#### Kisspeptin powder particle size characterisation

In anticipation that a powder formulation may provide a more stable alternative to solution preparations for use in the clinic, we also characterised the particle size and shape of the kisspeptin-54 powder using static automated imaging technology. The kisspeptin-54 powder was formed of irregular-shaped particles (Fig. 4b–c) which dispersed easily. The powder was characterised as having particles largely within the size range 10–110 μm, similar to those reported for successful commercial powder inhalers.^74^ This complies with the U.S. Food and Drug Administration^75^ and European Medicines Agency^76^ guidelines on the pharmaceutical quality of inhalation and nasal products which require control of particle size distribution, i.e., that the vast majority of the particles are larger than 10 μm,^76^ with over 40% of particles in the size range 20–50 μm, which is regarded as optimal for nasal deposition.^36^

### Effects of intranasal administration of kisspeptin in mice

Given that a notable *extra-hypothalamic* population of GnRH neurons persists within the OB of adult humans and the recent identification of an olfactory-reproductive GnRH pathway,^37^ we next asked whether this *extra-hypothalamic* population of GnRH neurons may mediate the effects of intranasal kisspeptin-54 administration on gonadotropin release, by undertaking a series of rodent studies in adult C57BL/6J male mice.

#### Intranasal administration of kisspeptin robustly and dose-dependently stimulates LH release in male mice

**Fig. 5:**
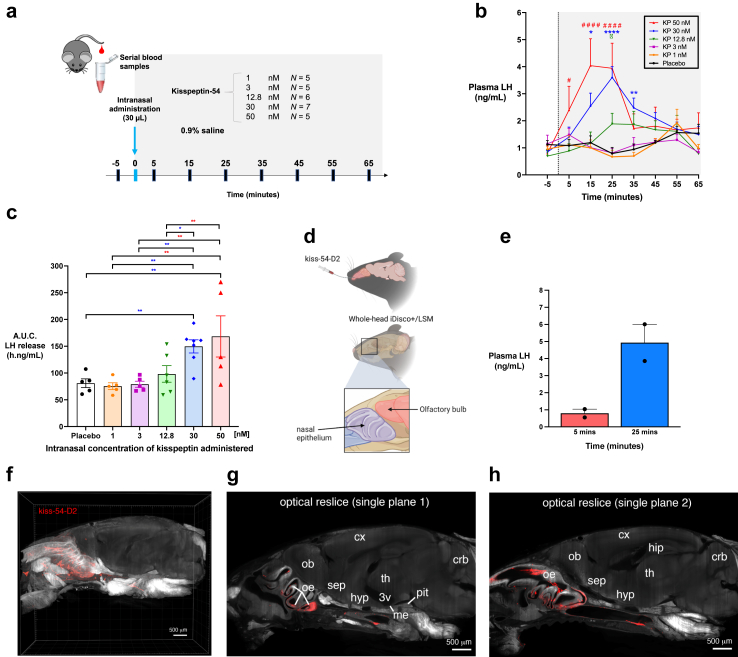
**Intranasal administration of kisspeptin stimulates LH release in a dose-dependent manner in adult male mice. (a)** Protocol schematic: A single volume of 30 μL of either 0.9% saline (placebo) or different concentrations of kisspeptin-54 (1, 3, 12.8, 30, or 50 nM solution; equivalent to 0.001–0.05 nmol/kg) was delivered into one nostril at timepoint 0 min. LH levels were assessed using tail-tip serial blood sampling, with the first sampling point at timepoint −5 min, followed by sampling every 10 min over 70 min. **(b)** Mean (±SEM) plasma LH (ng/mL) in adult male mice receiving intranasal administration of placebo or kisspeptin-54 at timepoint 0 min (dotted line). Shaded grey area indicates putative presence of the peptide in circulation. Group analysis revealed the presence of a dose-dependent effect of kisspeptin-54 on LH levels with a pronounced induction of LH secretion by 12 nM (^Φ^*P* < 0.05), 30 nM (∗∗∗∗*P* < 0.0001, ∗∗*P* < 0.01, ∗*P* < 0.05) and 50 nM (^####^*P* < 0.0001, ^#^*P* < 0.05) compared with 0.9% saline group levels (two-way ANOVA with Fisher’s LSD test). *N* = 5 per group except 12.8 nM where *N* = 6 and 30 nM where *N* = 7. **(c)** Integrated LH response to kisspeptin-54 is represented by area under the curve (A.U.C.) at different doses of kisspeptin-54. Groups were compared by one-way ANOVA with Fisher’s LSD test (∗∗*P* < 0.01, ∗*P* < 0.05). *N* = 5 per group except 12.8 nM where *N* = 6 and 30 nM where *N* = 7. **(d)** Experimental protocol schematic: Mice were injected intranasally with a fluorescently tagged kisspeptin-54 (kiss-54-D2) and sacrificed 25 min later to assess compound diffusion into the brain. After sacrifice and transcardial perfusion with 4% paraformaldehyde dissolved in PBS 0.01 M, pH 7.4, heads were removed and decalcified prior to whole-head tissue-clearing and light-sheet microscopy imaging. **(e)** Mean (±SEM) plasma LH (ng/mL) in adult male mice after intranasal administration of fluorescently-tagged kisspeptin-54 (kiss-54-D2) at 1 nMol. Intranasal kiss-54-D2 was administered at timepoint 0 min, with blood sampling for plasma LH at 5- and 25-min post-administration. *N* = 2. **(f)** Solvent-based tissue clearing, and light-sheet imaging revealed the distribution of kisspeptin-54-D2 (red) in a 3.5 mm-thick 3D sagittal projection of an optically cleared mouse head. **(g and h)**. Single-plane optical re-slices of the 3D volume facilitates precise visualisation and confirmed that kisspeptin-54-D2 remained restricted to the nasal compartment without reaching deeper brain structures. White = tissue auto-fluorescence, scale bars = 1 mm. 3v, third ventricle; crb, cerebellum; cx, cortex; hip, hippocampus; hyp, hypothalamus; me, median eminence; ob, olfactory bulb; oe, olfactory epithelium; pit, pituitary; sep, septum; th, thalamus.

It is well-established that *intraperitoneal* administration of kisspeptin-54 enhances LH secretion in male mice.^63^ However, to determine whether *intranasal* kisspeptin-54 administration can induce LH release similar to our aforementioned findings in humans, we first examined a range of different concentrations of kisspeptin-54 solution delivered intranasally to adult male mice (Fig. 5a). Following delivery of kisspeptin-54 or 0.9% saline (placebo) into one nostril, circulating LH levels were assessed every 10 min over 70 min. We observed that intranasal kisspeptin-54 administration at concentrations of 12.8–50 nM (0.0128–0.05 nmol/kg) significantly increased LH secretion over time (Fig. 5b). Time profile analysis identified that increasing the kisspeptin-54 concentration resulted in rapid increases in LH release with peak LH levels occurring at 25 min after intranasal administration for concentrations 12.8 nM at 1.9 ± 0.4 ng/mL (mean difference = 1.1 ng/mL [95% CI, 0.1–2.2], *P* = 0.047 vs. placebo, two-way ANOVA with Fishers LSD test) and 30 nM at 3.6 ± 0.4 ng/mL (mean difference = 2.8 ng/mL [95% CI, 1.8–3.9], *P* < 0.001 vs. placebo, two-way ANOVA with Fishers LSD test), compared to placebo administration (0.9 ± 0.2 ng/mL) (Fig. 5b). At the highest concentration of 50 nM, kisspeptin-54 promoted an LH peak level (4.0 ± 1.0 ng/mL, mean difference = 2.9 ng/mL [95% CI, 1.7–4.0], *P* < 0.001 vs. placebo, two-way ANOVA with Fishers LSD test) even more rapidly at 15 min when compared to the other doses evaluated. Analysis of the AUC LH response to kisspeptin-54 administration revealed that 30 nM is sufficient to reach peak LH secretion in mice as no statistical difference was observed in LH release with 50 nM (Fig. 5c and Supplemental Table S10). From a safety perspective, following intranasal kisspeptin-54 administration, we did not observe any adverse effects leading to pain, distress, or illness (i.e., our predetermined safety endpoints) within the first 72-h follow-up period. As such, mice were kept under the same housing conditions until the determined humane endpoint for our protocol.

#### Intranasal administration of fluorescently tagged kisspeptin accumulates in the olfactory epithelium in male mice

Because we observed that intranasal kisspeptin-54 administration triggers LH release in a dose-dependent manner in male mice (similar to humans), we next sought to investigate the candidate mechanism by which intranasally administered kisspeptin-54 may induce LH secretion. To explore this, we first tagged kisspeptin-54 with a fluorescent dye (kisspeptin-54-D2) and subsequently administered kisspeptin-54-D2 (30 μL; 12.8 nM) intranasally into adult C57BL/6J male mice (Fig. 5d). Since intranasal administration of kisspeptin-54 was shown to result in peak LH levels at 25 min (Fig. 5b) we assessed compound diffusion into the brain of animals 25 min post-administration. Using this approach, we identified the diffusion of kisspeptin-54-D2 in intact heads in 3D (Fig. 5f–h). At 25 min after intranasal administration, kisspeptin-54-D2 was restricted to the nasal compartment, with the highest concentration within the olfactory epithelium (Fig. 5f–h). This indicates that intranasally-administered kisspeptin-54 was taken up by the olfactory epithelium (but not beyond) in the same timeframe as the induction of gonadotropin secretion in male mice (Fig. 5e).

#### GnRH neurons located in the olfactory bulb of male mice express *Kiss1r* mRNA

**Fig. 6:**
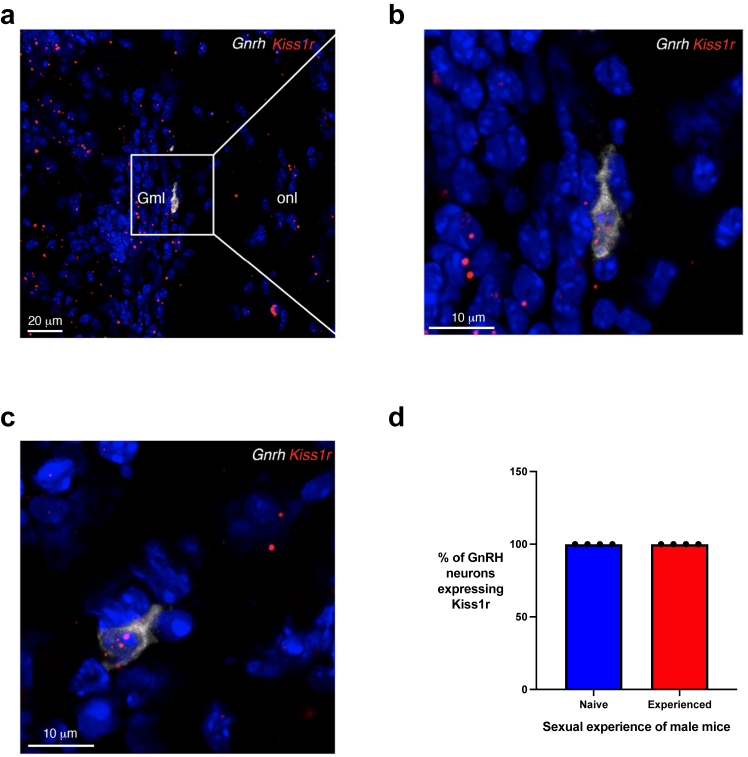
**GnRH neurons located in the olfactory bulb of male mice express *Kiss1r* mRNA. (a)** Fluorescent *in situ* hybridisation (RNAScope) for kisspeptin receptor mRNA (*Kiss1r*, red) and GnRH-1 mRNA (*Gnrh1*, white) shows *Kiss1r*-expressing GnRH neurons in the murine olfactory bulb of both naïve **(b)** and sexually experienced males **(c)**. Scale bars = 20 μm **(a)**; 10 μm **(b, c)**. **(a)** boxed area magnified in (**b)**. Gml, glomerular layer; onl, olfactory nerve layer. **(d)** Quantitative analysis showing that the entire population of GnRH neurons located in the olfactory bulb expresses *Kiss1r* in both naïve and sexually experienced males. *N* = 4.

Next, we sought to identify how the delivery of kisspeptin-54 to the olfactory epithelium could induce gonadotropin secretion. To this end, previous work has demonstrated an extra-hypothalamic population of GnRH neurons located in the OB of mice and humans^77^ and which could present hypophysiotropic properties in humans.^78^ Furthermore, very recent anatomical and functional data has shown that these GnRH neurons in the OB of adult male mice extend neurites into the olfactory epithelium and project to the hypothalamic regions modulating the secretion of downstream reproductive hormones.^37^ We therefore hypothesised that these GnRH neurons in the OB may express the kisspeptin receptor, which could permit intranasal kisspeptin delivery to stimulate GnRH neurons and so downstream reproductive hormone secretion. To explore this, we performed fluorescent *in situ* hybridisation using specific probes for *Kiss1r* and *Gnrh1* in male mice (Fig. 6a–c), visualising *Kiss1r* expression in GnRH neurons located in the OB (Fig. 6a–c).

Since sexual experience is known to affect gene expression in the rodents’ brain, we performed similar experiments in both sexually naïve and sexually experienced males. Quantitative analysis showed that 100% of OB-GnRH neurons express *Kiss1r* mRNA in male mice regardless of their sexual experience (Fig. 6d). Taken together, this suggests that *extra-hypothalamic* olfactory GnRH neurons are the direct targets of intranasal kisspeptin, driving its effects on gonadotropin release through a GnRH olfactory-reproductive pathway.

## Discussion

Herein, we demonstrate for the first time that intranasal kisspeptin delivery robustly stimulates gonadotropin release in healthy participants and patients with reproductive disorders. These data will further drive the escalating development of kisspeptin therapeutics for the management of common reproductive disorders as they identify the first non-invasive route for kisspeptin administration that would be preferable to patients and clinicians and so be potentially practice-changing in the field.

As kisspeptin had not previously been administered via the intranasal route, we first evaluated a broad range of doses between 3.2 and 25.6 nmol/kg of kisspeptin-54 in healthy men, based on previous human studies of subcutaneous kisspeptin-54 administration.^16^ Here, intranasal delivery of kisspeptin to healthy men resulted in clinically-significant increases in serum LH and in a dose-dependent manner with the highest levels occurring 30–45 min post-administration, and crucially without any side effects. While a dose-related increase in serum FSH was observed, kisspeptin had a greater stimulatory effect on LH release, which is consistent with previous studies in humans using the intravenous and subcutaneous route.^54^^,^^56^, ^57^, ^58^ Following intranasal kisspeptin administration, there was a clinically-significant increase in serum testosterone following the 12.8 nmol/kg dose.

It is interesting to compare the pharmacodynamic profile of intranasal kisspeptin delivery with previous (invasive) administration routes. We have previously examined a single intravenous bolus of 6.4, 12.8, and 25.6 nmol/kg of kisspeptin-54 in healthy men.^59^ In that study, peak LH levels occurred 4–6 h after *intravenous* kisspeptin administration with a maximal increase from baseline of 8–10 IU/L for the three doses tested.^59^ In the current study, a far more rapid increase in serum LH was observed after *intranasal* kisspeptin for all four doses tested with peak LH stimulation (4.45 IU/L above baseline for 12.8 nmol/kg) within 30–45 min post-administration. This is consistent with the rapid onset of action with other intranasally delivered medications with direct brain-targeted delivery.^79^^,^^80^ Thus, the faster gonadotropin responses following intranasal compared to intravenous may suggest that intranasal kisspeptin administration capitalises on the aforementioned recently characterised olfactory-reproductive pathway.^37^ Moreover, the finding that the GnRH neuronal population in the OB although remarkably large (20% of entire rodent GnRH population) is smaller than the hypothalamic population,^37^ likely contributes to the smaller gonadotropin response following intranasal compared to intravenous kisspeptin administration.

In the current study, the increase in serum LH following 25.6 nmol/kg of kisspeptin-54 was lower than the rise observed with 12.8 nmol/kg. Similarly, in our previous work in healthy men, a single intravenous bolus of 25.6 nmol/kg resulted in less stimulation in LH release than 12.8 nmol/kg.^59^ It is intriguing to speculate about the reasons for this ceiling effect. Administration of higher single or cumulative doses of kisspeptin can result in desensitisation of kisspeptin receptors due to tachyphylaxis and hence a reduction in gonadotropins. This is mostly encountered in the context of frequent high-dose administration.^9^, ^10^, ^11^ Thus, further studies are warranted to determine the optimal doses and dosing frequency to elicit a sustained rise in gonadotropins but avoid tachyphylaxis as can occur with chronic intravenous/subcutaneous administration.

Given our demonstration that intranasal administration of kisspeptin robustly and dose-dependently stimulated gonadotropin release in healthy men, we wanted to determine whether this could be recapitulated in women and have therapeutic relevance to reproductive disorders. To explore this, we investigated both healthy women and patients with a common reproductive disorder in whom kisspeptin administration has shown promising clinical effects, namely women with HA.^4^^,^^9^, ^10^, ^11^ We show that intranasal kisspeptin given at the most efficacious dose in healthy men (12.8 nmol/kg) robustly stimulated gonadotropin release. Remarkably, despite being administered with the same dose of kisspeptin-54, the maximal LH and FSH increases were over 3-fold and 10-fold more potent, respectively, in the patients with HA in comparison with the healthy women. Indeed, this heightened response is consistent with previous clinical data comparing gonadotropin stimulation following subcutaneous kisspeptin administration in healthy women and women with HA.^4^ From a mechanistic perspective, although in healthy women kisspeptin responsiveness is dependent on ambient oestradiol levels (which are low in HA),^56^ it is well-established that *Kiss1r* expression is markedly increased (by approximately ∼2-fold) in the hypothalamus of female rodents under conditions of negative energy balance (akin to HA).^81^ Therefore, our findings may be explained by a greater abundance of KISS1R in HA, resulting in an exaggerated gonadotropin response to kisspeptin administration in the patients with HA compared with the healthy women.

During the 4-h acute time-course, we did not observe a significant increase in downstream oestradiol levels in women (although we did observe a significant testosterone increase in men). Regarding this, our previous data suggest that at least 4 h are required for serum sex steroid levels to peak after *subcutaneous* kisspeptin administration in women.^9^^,^^10^^,^^56^ Indeed, in the current study, circulating serum oestradiol continued to rise throughout the sampling period in the patients with HA, suggesting that a longer study duration may have revealed more pronounced alterations in sex steroid secretion warranting further study. Moreover, ovarian oestrogen synthesis is not immediate and depends on follicle growth and development, involving both the granulosa and theca cells found in ovarian follicles.^82^ Specifically, theca cells are not capable of producing oestrogen but do produce androgens in response to LH, which are subsequently converted into oestrogen by FSH-induced aromatase in granulosa cells.^83^ Therefore, based on our promising results of significant gonadotropin release with a reassuring safety-profile, further chronic studies are now warranted to determine whether repeated doses can translate into increases in downstream sex steroid levels as seen with *subcutaneous* kisspeptin administration,^9^^,^^10^^,^^84^ as well as improved clinical outcomes in patients with HA, such as restoration of menstrual cyclicity. Additionally, studies exploring the clinical benefits of intranasal kisspeptin in patients with other common reproductive disorders would be of immediate clinical interest.

Better understanding of the mechanisms of drug transfer to the brain will enable delivery devices and drug formulations to be optimised for delivery and uptake at the relevant site(s) of absorption within the nasal cavity. Nasal deposition was evaluated using a recently developed nasal cavity replica, AINI, which is a benchtop test apparatus representing an idealised geometry derived from the CT scans of 12 individual adults. This sectioned cast was designed specifically to guide the development and evaluate the performance of nasal drug delivery systems by estimating *in vivo* regional nasal cavity deposition patterns.^60^^,^^85^ Using this experimental approach, following administration of nasal sprays containing kisspeptin, we demonstrated that regional deposition was reproducible with approximately 70% of the spray delivered beyond the nasal vestibule with the majority depositing in the turbinates. This finding is highly relevant given that a significant proportion of the olfactory epithelium in humans is situated on the turbinates, with neuronal connections to the OB^86^^,^^87^ coupled with our demonstration in rodents that intranasal administration of fluorescently-tagged kisspeptin accumulates in the olfactory epithelium in the same timeframe as the induction of gonadotropin secretion.

To advance intranasal kisspeptin therapeutics through the translational pathway, it is vital to consider the viability of developing kisspeptin as a medicine. Thus, we evaluated the chemical stability of kisspeptin-54 in 0.9% saline.^73^ Crucially from a product quality perspective, kisspeptin-54 was stable in solution when stored at 4 °C (typical home refrigerator temperature) for approximately 2 months, without the use of stabilisers to optimise the formulation. Alternatively, if kisspeptin-54 stability in solution is a limiting factor, the powder form appears to be amenable to nasal insufflation. Indeed, from a product development perspective, kisspeptin receptor agonists have also been developed which display enhanced potency and longer duration of action than native kisspeptin when administered by subcutaneous injection^4^ and so merit investigation using the intranasal route. Taken together, our pharmaceutical data suggest that it is feasible to develop a kisspeptin medical product for nasal delivery (in solution or powder) that is easily self-administered by patients at home, similar to the well-established clinically used intranasal desmopressin for diabetes insipidus (arginine vasopressin deficiency).

Having shown that intranasal kisspeptin induces dose-dependent gonadotropin release in humans, we finally conducted a series of rodent experiments to identify a putative mechanism for how intranasal kisspeptin may be mediating its effects. To this end, we demonstrate that intranasal administration of kisspeptin-54 (as well as fluorescently-tagged kisspeptin-54) induced a robust LH increase at 25 min post administration, recapitulating the findings in humans where we observed an increase within the same timeframe, suggesting that there may be a common mechanism in the mediation of the signal. Using intranasal delivery of fluorescently tagged kisspeptin-54, we demonstrated the accumulation of fluorescently-tagged kisspeptin in the olfactory epithelium, as well as crucially the presence of kisspeptin receptors in GnRH neurons in the OB. Taken together with very recent evidence of anatomical and functional connectivity between the olfactory epithelium and the hypothalamic preoptic area and median eminence via a plentiful *extra-hypothalamic* GnRH neuron population in the OB of adult mice,^37^ our data suggest that intranasal kisspeptin administration may capitalise on this olfactory-reproductive pathway. Specifically, previous rodent data identifies that stimulation of GnRH neurons in the OB influences the firing frequency of *hypothalamic* GnRH neurons in the preoptic area, primarily by amplifying glutamatergic signalling to hypothalamic GnRH neurons and ultimately resulting in downstream reproductive hormone secretion.^37^ Altogether, this suggests that intranasal kisspeptin administration may target these *extra-hypothalamic* OB-GnRH neurons, whose excitation is subsequently transmitted to *hypothalamic* GnRH neurons triggering a reproductive neuroendocrine response by the established *hypothalamic* GnRH neuronal system. Moreover, it is significant that the nasal cavity is highly rich in vasculature, which permits *some* intranasally administered medications to be absorbed by abundant local blood vessels and enter systemic circulation.^88^ Therefore, an alternative mechanism in the pathway mediating intranasal kisspeptin’s effects on reproductive hormones beyond via *extra-hypothalamic* OB-GnRH neurons could also be through systemic blood circulation. From here, intranasal kisspeptin may elicit a reproductive hormonal response similar to that following peripheral (intravenous) administration.^71^^,^^89^ Of note, the markedly faster onset of LH increase following *intranasal* kisspeptin administration compared to *intravenous* kisspeptin administration^59^ supports a unique and predominant action of intranasal kisspeptin directly on OB-GnRH neurons and so elucidating the relative contributions of each pathway warrants further study.

It is important to consider the strengths and weaknesses of the clinical studies. The strengths included its randomised, double-blinded, placebo-controlled design, as well as standardised assessments and procedures. The crossover design, in which the participants acted as their own controls, thereby minimised variability and enhanced power. All studies commenced in the morning to ensure peak basal reproductive hormone levels. Regarding the healthy women, study visits were matched in terms of the menstrual cycle phase and were all conducted in the follicular phase. To reduce variability in absorption, participants were familiarised with the procedure of intranasal application and were provided with written administration instructions prior to each study visit and self-administered the interventions under supervision from the clinical team. From a feasibility perspective, participants were easily accustomed to the procedure of intranasal administration within a few minutes of education, making it a practical delivery route for patients at home. The study also benefited from full compliance with all participants completing the protocol, i.e., five study visits in healthy men and two study visits in healthy women and patients with reproductive disorders (including a placebo visit per participant) and no missing data. Furthermore, the study was adequately powered with the sample size typical of proof-of-concept studies^55^ and compared favourably with our previous work examining the effects of intravenous and subcutaneous kisspeptin administration on reproductive hormone secretion.^4^^,^^54^^,^^56^, ^57^, ^58^ This enabled us to reveal significant stimulation in gonadotropin release following kisspeptin administration.

Regarding limitations, the study visits in healthy women were all conducted in the phase of the menstrual cycle when kisspeptin stimulates gonadotropin release the least (i.e., the follicular phase).^56^ However, patients with HA display a reproductive hormone profile that resembles the follicular phase (i.e., low circulating gonadotropin and oestradiol levels) more closely than the other phases, allowing us to compare kisspeptin’s response in the two cohorts of women more accurately. Therefore, an interesting area for future study would be examining the reproductive hormone profiles after intranasal kisspeptin administration in healthy women during the preovulatory and luteal phases when kisspeptin is more potent and so may result in even greater reproductive hormone stimulation.^56^ Furthermore, it should be noted that although all participants received standardised administration instructions, it is possible that the participants for whom the reproductive hormones did not increase to the same level as other participants in the same dosing arm, may have used poor nasal spray administration technique, resulting in less effective kisspeptin delivery. Furthermore, it is important to note that our mechanistic studies were performed in (male) rodents. However, it is well-recognised that the rodent and human olfactory systems share several common characteristics (including the basic organisation of the olfactory central nervous system), meaning that rodents provide a robust model for studying the olfactory system in men and women.^90^ Finally, in our rodent studies, we measured LH secretion (as an established biomarker for the effect of kisspeptin on GnRH secretion), but not FSH and testosterone levels. However, these would be expected to increase in response to intranasal kisspeptin delivery, in line with the effects observed in our human clinical study.

Our demonstration that intranasal kisspeptin delivery rapidly stimulates gonadotropin release raises the possibility that this novel property can be exploited therapeutically or diagnostically. For instance, in addition to the classical reproductive axis, emerging data demonstrates that kisspeptin also modulates sexual behaviour.^91^^,^^92^ Indeed, we have recently demonstrated that intravenous kisspeptin administration to women^27^ and men^28^ with low sexual desire robustly enhances sexual brain processing with associated improvements in sexual desire and arousal and a pro-erectile effect in men. Of clinical significance, the experimental protocol employed in these studies resulted in a similar clinically-significant mean maximal rise in serum LH^27^^,^^28^ to what we observed in the current study with the 12.8 nmol/kg dosing. Given that this increment is associated with behavioural measures, it would therefore be attractive to investigate whether intranasal delivery could be an effective kisspeptin administration route for the management of psychosexual disorders and also capitalise on recently identified additional direct olfactory-limbic neuronal connections.^37^ Finally, beyond therapeutics, kisspeptin also has diagnostic potential for differentiating common causes of delayed puberty^71^ and menstrual disturbance in women.^93^ Therefore, intranasal kisspeptin offers not only a novel route of administration for the management of reproductive and psychosexual disorders but also a potential simple non-invasive diagnostic test to interrogate hypothalamic function in patients with reproductive disorders.

In summary, we provide the first human evidence identifying intranasal delivery as a novel, non-invasive, and well-tolerated route of kisspeptin administration to rapidly and robustly stimulate gonadotropin release. Furthermore, the demonstration that kisspeptin-54 in 0.9% saline solution remains within pharmaceutically accepted limits for stability for up to 60 days at 4 °C, provides a realistic opportunity to create a patient self-administered nasal medicine that would be preferable to current invasive methods (injection) for patients and clinicians alike. To this end, our data support further evaluation of intranasal kisspeptin administration in larger-scale clinical studies.

## Contributors

Conceptualisation/Methodology: EGM, BF, VP, KC, PG, ANC, WSD.

Funding acquisition: EGM, ANC, WSD.

Data collection: EGM, MSBS, VD, LD, GT, JT, LT, MP, BP, LY, SAC, MY, ECA, SN, AY, MC, AN, MS.

Data analysis: EGM, MSBS, VD, PB, AA, MS, BF, VP, KC, PG, ANC, WSD.

Supervision: BF, VP, KC, PG, ANC, WSD.

Manuscript: EGM, MSBS, BF, VP, KC, PG, ANC, WSD.

The first author (EGM) and last two authors (ANC and WSD) had unrestricted access to the data after the database was locked, wrote the manuscript (all other authors reviewed, amended, and approved the manuscript), made the decision to submit the manuscript for publication, and vouch for the completeness and accuracy of the data and for the adherence of the study to the protocol. All authors read and approved the final version of the manuscript.

## Data sharing statement

Some data sets generated during and/or analysed during the present study are not publicly available but are available from the corresponding authors based on reasonable scientific merit. All data provided are anonymised to respect the privacy of the participants.

## Declaration of interests

AA has conducted consultancy work for Myovant Sciences Ltd outside the submitted work. WSD has conducted consultancy work for AbCellera, PostEra, KaNDy Therapeutics Ltd, and Myovant Sciences Ltd outside the submitted work. The other authors declare no conflict-of-interest.

## Appendix ASupplementary data

Supplemental Figs. S1–S5 and Tables S1–S10

Intranasal Kisspeptin Protocol
