# Supplementary material for: Intranasal kisspeptin administration rapidly stimulates gonadotropin release in humans
Source: eBioMedicine. 2025 Apr 11;115:105689. doi: 10.1016/j.ebiom.2025.105689 (PMC12018048; doi:10.1016/j.ebiom.2025.105689)
Supplement: Supplemental Figs. S1–S5 and Tables S1–S10 [file mmc1.docx]

**Supplemental: Intranasal Kisspeptin Administration
Rapidly Stimulates Gonadotropin Release in Humans**

**Authors:**

Edouard G. Mills^1,2^, Mauro SB. Silva^3+^, Virginia Delli^3+^, Laurine Decoster^3^, Gaetan Ternier^3^, Jovanna Tsoutsouki^1^, Layla Thurston^1^, Maria Phylactou^1,2^, Bijal Patel^1^, Lisa Yang^1^, Sophie A. Clarke^1^, Megan Young^1^, Emma C. Alexander^1^, Sandhi Nyunt^1^, Arthur C. Yeung^1^, Muhammad Choudhury^1^, Anastasia Newman^1^, Paul Bech^1^, Ali Abbara^1^, Magda Swedrowska^4^, Ben Forbes^4^, Vincent Prévot^3^, Konstantina Chachlaki^3^, Paolo Giacobini^3^, Alexander N. Comninos^1,2^*, Waljit S. Dhillo^1,2^*

^+^ These authors contributed equally. * Co-senior, co-corresponding authors.

**Affiliations:**

^1^Section of Endocrinology and Investigative Medicine, Imperial College London, London, UK.

^2^Department of Endocrinology, Imperial College Healthcare NHS Trust, London, UK.

^3^Laboratory of Development and Plasticity of the Neuroendocrine Brain, Lille Neuroscience and Cognition, Univ. Lille, Inserm, CHU Lille, Lille, France.
^4^Institute of Pharmaceutical Science, King’s College London, London, UK.

**Correspondence to:**

Professors Alexander N. Comninos & Waljit S. Dhillo,

Section of Endocrinology and Investigative Medicine,

Imperial College London,

6^th^ Floor, Commonwealth Building, Hammersmith Hospital Campus,

Du Cane Road, London W12 0NN, United Kingdom.

+442075943487 [a.comninos@imperial.ac.uk](mailto:a.comninos@imperial.ac.uk) [w.dhillo@imperial.ac.uk](mailto:w.dhillo@imperial.ac.uk)

**Supplemental Figure 1: Participant recruitment and flow summary for the clinical study in healthy men.**

**
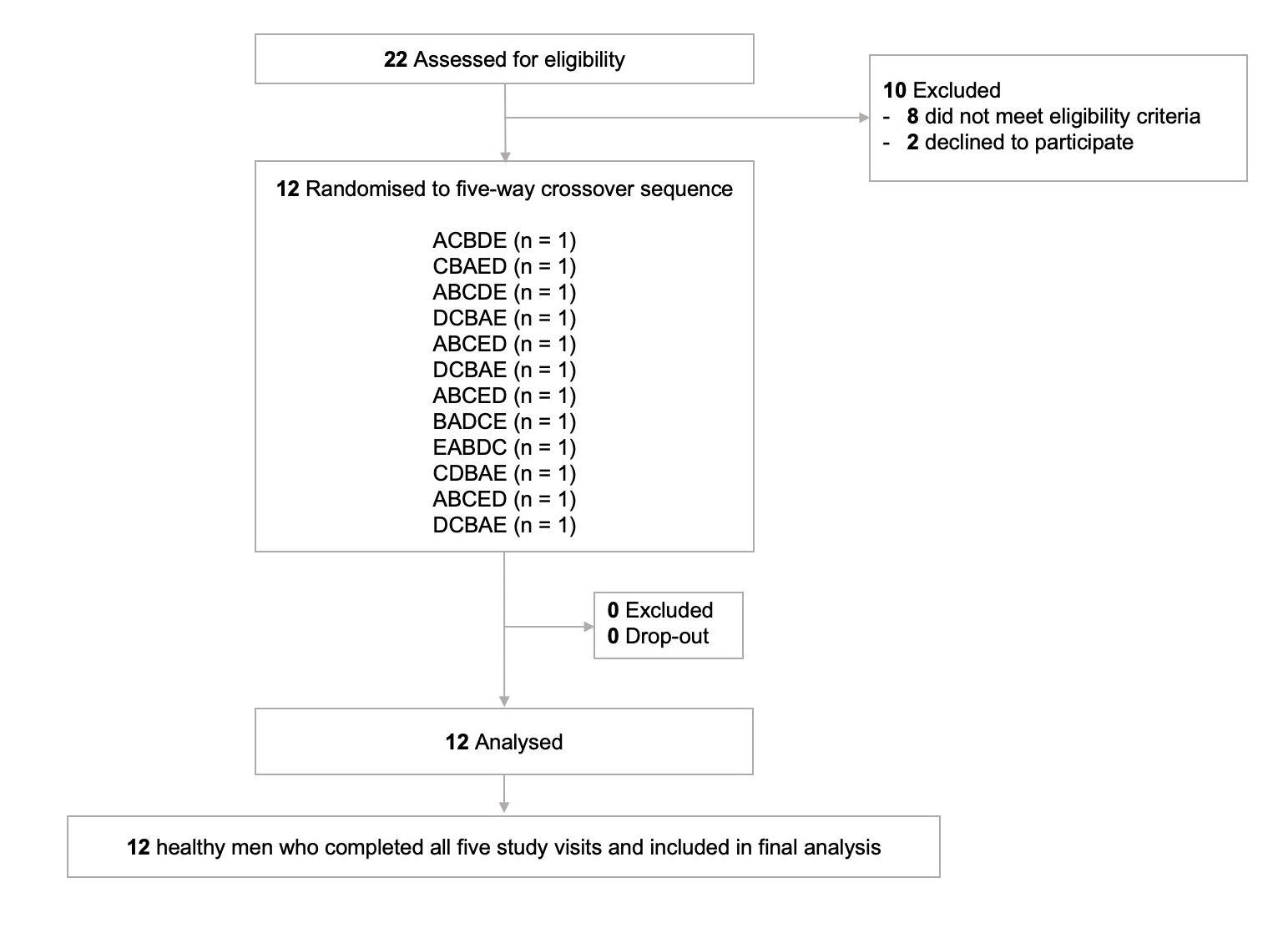
**

Following screening and informed consent, 12 healthy men completed five study visits each, receiving the following five interventions in random order via the intranasal route: 3.2, 6.4, 12.8, and 25.6 nmol/kg of kisspeptin-54, and 0.9% saline (placebo). Study visits were separated by a minimum of one week.

Abbreviations: A, 3.2 nmol/kg of kisspeptin-54; B, 6.4 nmol/kg; C, 12.8 nmol/kg; D, 25.6 nmol/kg; E, placebo.

**Supplemental Figure 2: Participant recruitment and flow summary for the clinical study in healthy women.**

**
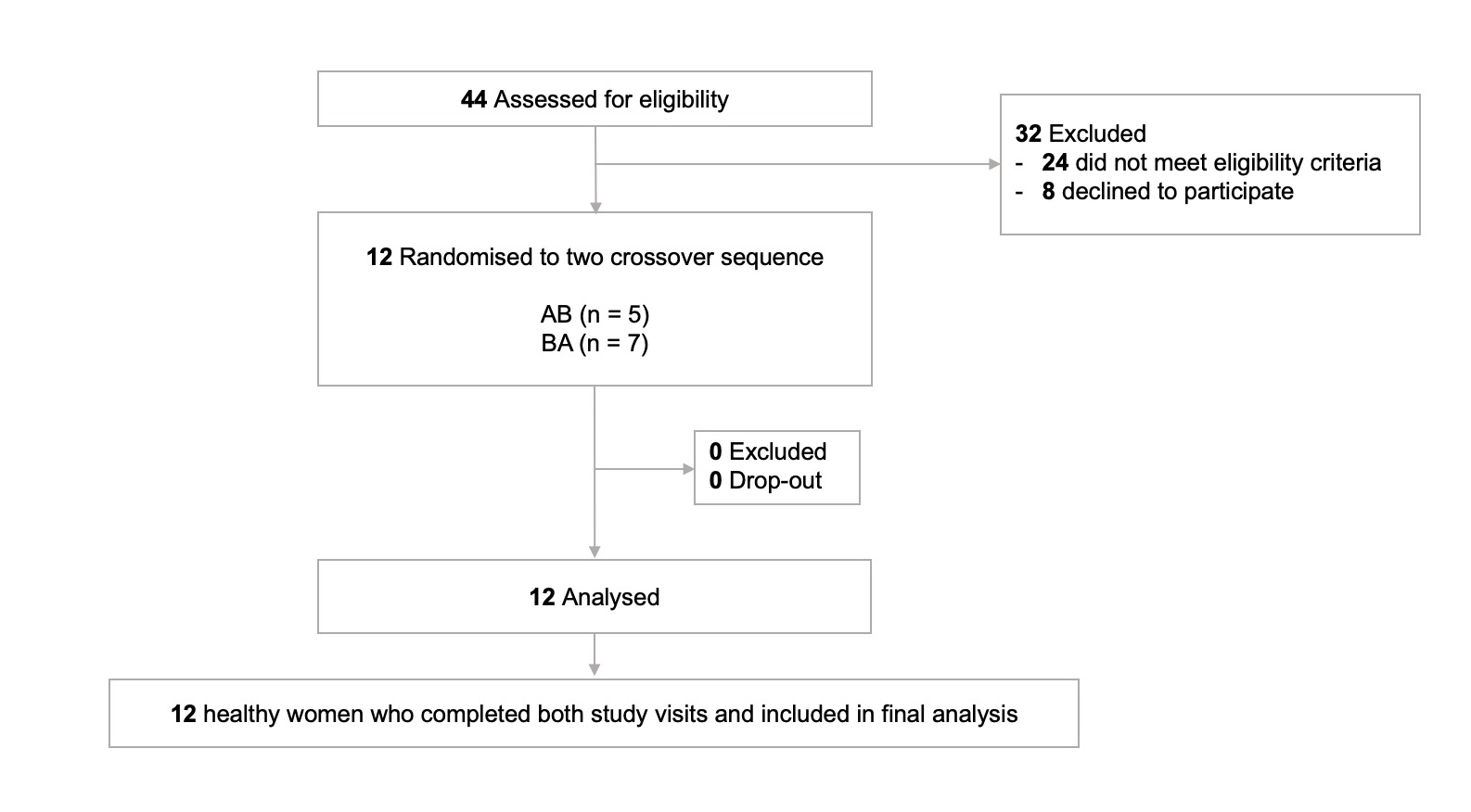
**

Following screening and informed consent, 12 healthy women completed two study visits each, receiving the following two interventions in random order via the intranasal route: 12.8 nmol/kg of kisspeptin-54, and 0.9% saline (placebo). Study visits were conducted in the follicular phase of the menstrual cycle, with one intervention administered per menstrual cycle.

Abbreviations: A, 12.8 nmol/kg of kisspeptin-54; B, placebo.

**Supplemental Figure 3: Effects of intranasal administration of kisspeptin in healthy women.**


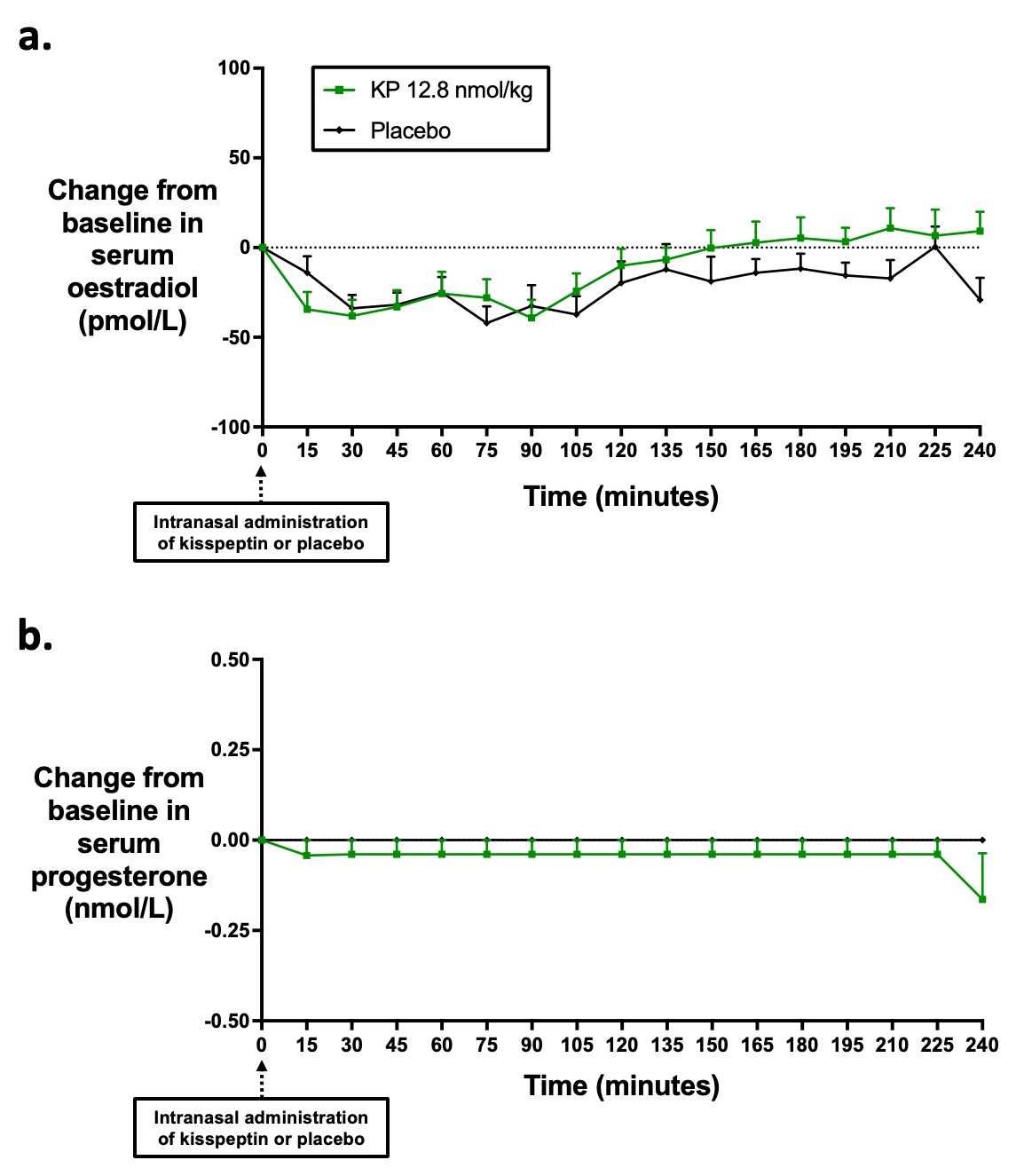


**(a and b)**: Mean (± SEM) change from baseline in serum oestradiol (pmol/L) **(a)** and serum progesterone (nmol/L) **(b)** in healthy women receiving intranasal administration of kisspeptin-54 or placebo at timepoint 0 minutes. Groups were compared by two-way ANOVA with post-hoc Bonferroni multiple comparison test. *N* = 12.

**Supplemental Figure 4: Participant recruitment and flow summary for the clinical study in patients with hypothalamic amenorrhoea.**

**
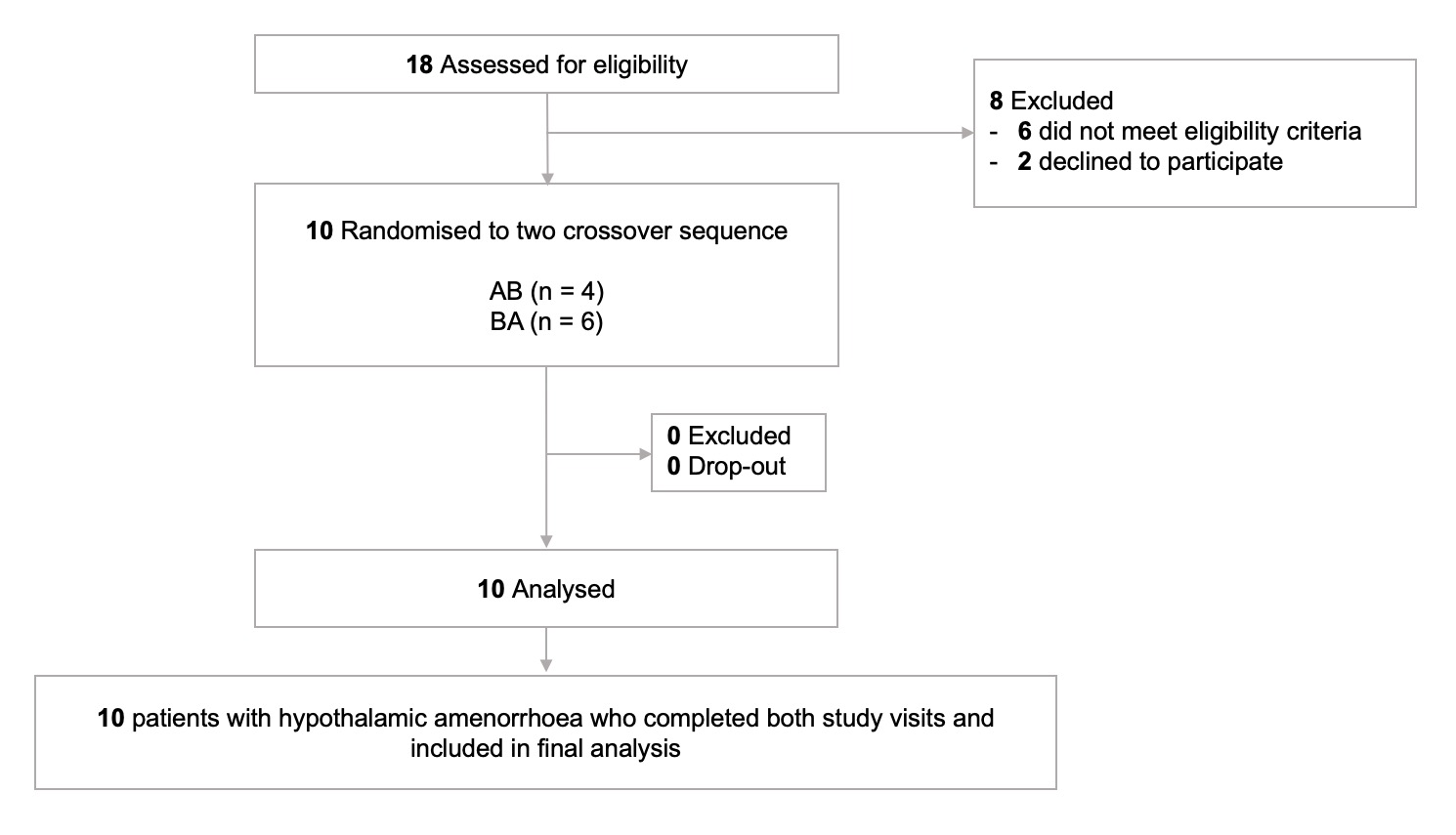
**

Following screening and informed consent, 10 patients with hypothalamic amenorrhoea completed two study visits each, receiving the following two interventions in random order via the intranasal route: 12.8 nmol/kg of kisspeptin-54, and 0.9% saline (placebo). Study visits were separated by a minimum of one week.

Abbreviations: A, 12.8 nmol/kg of kisspeptin-54; B, placebo.

**Supplemental Figure 5: Effects of intranasal administration of kisspeptin in patients with hypothalamic amenorrhoea.**


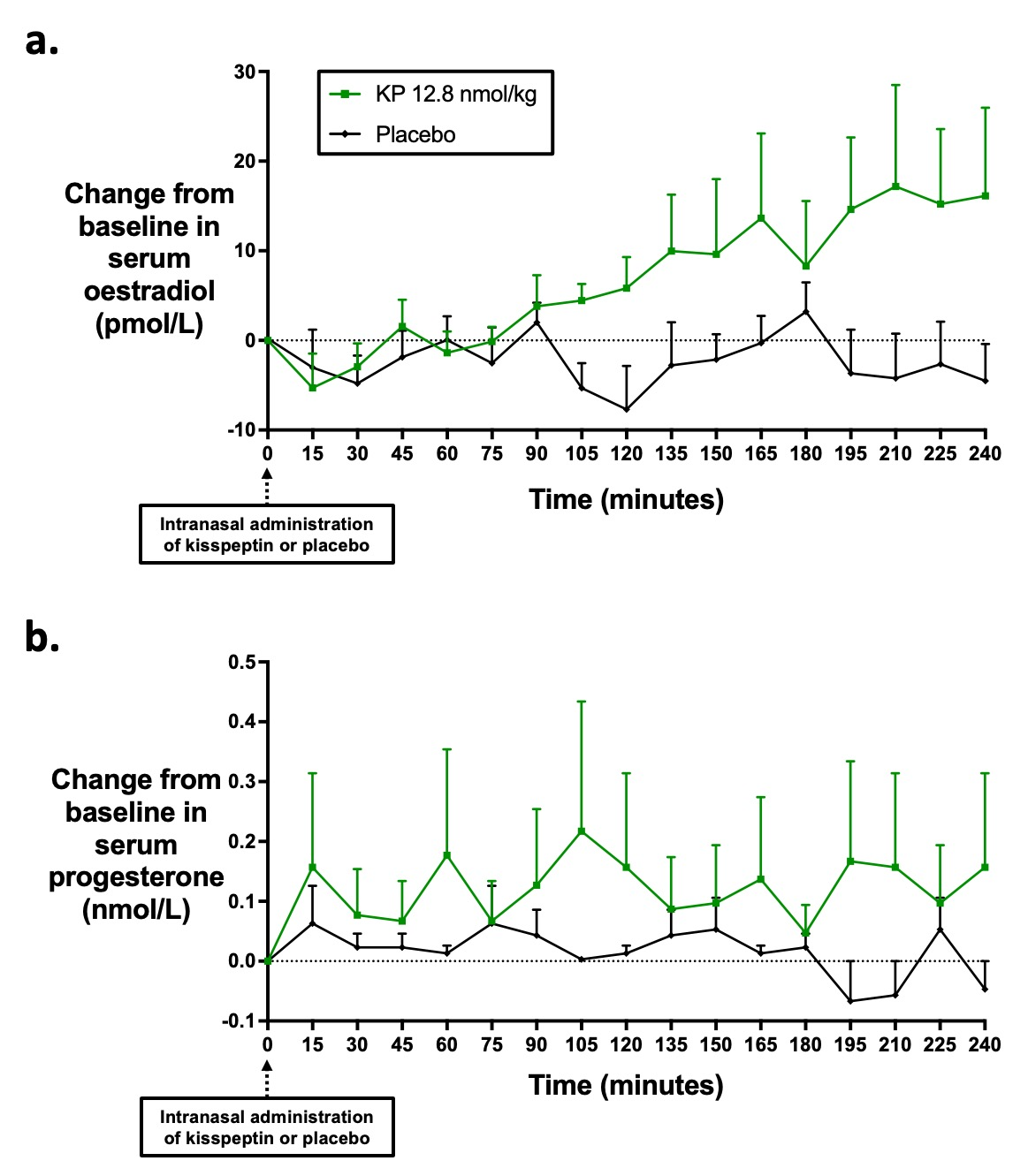


**(a and b)**: Mean (± SEM) change from baseline in serum oestradiol (pmol/L) **(a)** and serum progesterone (nmol/L) **(b)** in patients with hypothalamic amenorrhoea receiving intranasal administration of kisspeptin-54 or placebo at timepoint 0 minutes. Groups were compared by two-way ANOVA with post-hoc Bonferroni multiple comparison test. *N* = 10.

**Supplemental Table 1: Baseline clinical characteristics at each study visit in healthy men.**

|  |  | **Kisspeptin**  **3.2 nmol/kg**  **(*N* = 12)** | **Kisspeptin**  **6.4 nmol/kg  (*N* = 12)** | **Kisspeptin 12.8 nmol/kg (*N* = 12)** | **Kisspeptin 25.6 nmol/kg (*N* = 12)** | **Placebo**  **(0.9% saline)**  **(*N* = 12)** |
| --- | --- | --- | --- | --- | --- | --- |
| **Baseline Reproductive Hormones** |  |  |  |  |  |  |
|  | LH (IU/L) | 2.98 ± 0.27 | 2.88 ± 0.22 | 2.83 ± 0.21 | 3.04 ± 0.41 | 3.07 ± 0.33 |
|  | FSH (IU/L) | 3.44 ± 0.79 | 3.37 ± 0.82 | 3.35 ± 0.72 | 3.48 ± 0.76 | 3.53 ± 0.85 |
|  | Testosterone (nmol/L) | 16.64 ± 1.42 | 18.01 ± 1.56 | 17.85 ± 1.47 | 17.34 ± 1.83 | 17.45 ± 1.68 |

Mean ± SEM are presented. FSH, follicle-stimulating hormone; LH, luteinising hormone.

**Supplemental Table 2: Intranasal administration of kisspeptin stimulates reproductive hormone secretion in healthy men.**

| **Reproductive Hormone** | | **Intranasal Intervention** | **Mean**  **± SEM** | **Mean difference** | **95% CI** | ***P-*Value** |
| --- | --- | --- | --- | --- | --- | --- |
| **LH** |  |  |  |  |  |  |
|  | **AUC (h·IU/L)** |  |  |  |  |  |
|  |  | Placebo | -25.4 ± 70.5 | ---- | ---- | ---- |
|  |  | Kisspeptin 3.2 nmol/kg | 172.2 ± 64.2 | 197.6 | 10.6 to 384.6 | ***P* = 0.037*** |
|  |  | Kisspeptin 6.4 nmol/kg | 300.2 ± 79.2 | 325.5 | 122.6 to 528.4 | ***P* = 0.002**** |
|  |  | Kisspeptin 12.8 nmol/kg | 595.7 ± 98.3 | 621.1 | 257.2 to 985.1 | ***P* = 0.001**** |
|  |  | Kisspeptin 25.6 nmol/kg | 549.0 ± 108.6 | 574.4 | 353.7 to 795.1 | ***P* < 0.001****** |
|  | **Maximum increase from baseline (IU/L)** | |  |  |  |  |
|  |  | Placebo | 1.4 ± 0.3 | ---- | ---- | ---- |
|  |  | Kisspeptin 3.2 nmol/kg | 2.5 ± 0.3 | 1.1 | 0.2 to 2.0 | ***P* = 0.015*** |
|  |  | Kisspeptin 6.4 nmol/kg | 3.1 + 0.5 | 1.7 | 0.4 to 3.0 | ***P* = 0.012*** |
|  |  | Kisspeptin 12.8 nmol/kg | 4.4 ± 0.6 | 3.1 | 1.2 to 4.9 | ***P* = 0.002**** |
|  |  | Kisspeptin 25.6 nmol/kg | 4.3 ± 0.6 | 2.9 | 1.6 to 4.1 | ***P* < 0.001***** |
| **FSH** |  |  |  |  |  |  |
|  | **AUC (h·IU/L)** |  |  |  |  |  |
|  |  | Placebo | -60.3 ± 27.5 | ---- | ---- | ---- |
|  |  | Kisspeptin 3.2 nmol/kg | -13.1 + 23.1 | 47.2 | -0.8 to 95.2 | *P* = 0.055 |
|  |  | Kisspeptin 6.4 nmol/kg | 37.3 ± 16.2 | 97.6 | -9.9 to 205.1 | *P* = 0.082 |
|  |  | Kisspeptin 12.8 nmol/kg | 96.3 ± 23.2 | 156.6 | 14.2 to 299.0 | ***P* = 0.029*** |
|  |  | Kisspeptin 25.6 nmol/kg | 87.3 ± 66.2 | 147.6 | -100.8 to 396.0 | *P* = 0.417 |
|  | **Maximum increase from baseline (IU/L)** | |  |  |  |  |
|  |  | Placebo | 0.1 ± 0.0 | ---- | ---- | ---- |
|  |  | Kisspeptin 3.2 nmol/kg | 0.3 ± 0.1 | 0.2 | 0.1 to 0.4 | ***P* = 0.014*** |
|  |  | Kisspeptin 6.4 nmol/kg | 0.4 ± 0.1 | 0.4 | 0.1 to 0.7 | ***P* = 0.035*** |
|  |  | Kisspeptin 12.8 nmol/kg | 0.7 ± 0.2 | 0.7 | 0.1 to 1.3 | ***P* = 0.022*** |
|  |  | Kisspeptin 25.6 nmol/kg | 0.8 ± 0.3 | 0.7 | -0.2 to 1.6 | *P* = 0.133 |
| **Testosterone** | |  |  |  |  |  |
|  | **AUC (h·nmol/L)** |  |  |  |  |  |
|  |  | Placebo | -183.8 ± 201.4 | ---- | ---- | ---- |
|  |  | Kisspeptin 3.2 nmol/kg | 273.6 ± 158.7 | 457.4 | -27.5 to 942.3 | *P* = 0.068 |
|  |  | Kisspeptin 6.4 nmol/kg | 145.9 ± 158.5 | 329.7 | -161.7 to 821.2 | *P* = 0.283 |
|  |  | Kisspeptin 12.8 nmol/kg | 470.4 ± 144.0 | 654.3 | 224.6 to 1084.0 | ***P* = 0.003**** |
|  |  | Kisspeptin 25.6 nmol/kg | 446.5 ± 287.4 | 630.3 | -38.8 to 1299.0 | *P* = 0.068 |
|  | **Max increase from baseline (nmol/L)** | |  |  |  |  |
|  |  | Placebo | 2.5 ± 0.7 | ---- | ---- | ---- |
|  |  | Kisspeptin 3.2 nmol/kg | 4.0 ± 1.0 | 1.5 | -1.1 to 4.1 | *P* = 0.460 |
|  |  | Kisspeptin 6.4 nmol/kg | 3.4 ± 0.8 | 0.9 | -1.3 to 3.1 | *P* = 0.975 |
|  |  | Kisspeptin 12.8 nmol/kg | 4.9 ± 0.7 | 2.4 | 0.4 to 4.4 | ***P* = 0.018*** |
|  |  | Kisspeptin 25.6 nmol/kg | 5.6 ± 1.6 | 3.1 | -0.7 to 6.8 | *P* = 0.135 |

Mean (± SEM) area under the curve (AUC) of the change in serum luteinising hormone (LH) (h.IU/L), follicle-stimulating hormone (FSH) (h.IU/L), and testosterone (h.nmol/L), and mean (± SEM) maximum increase from baseline in serum LH (IU/L), FSH (IU/L), and testosterone (nmol/L). *P-*values represent comparison between the mean differences following intranasal kisspeptin administration vs. placebo. Groups were compared by one-way ANOVA with post-hoc Bonferroni multiple comparison test. **P* < 0.05, ***P* < 0.01, ****P* < 0.001, *****P* < 0.0001. *N* = 12.

**Supplemental Table 3: Baseline clinical characteristics at each study visit in healthy women.**

|  |  | **Kisspeptin**  **12.8 nmol/kg**  **(*N* = 12)** | **Placebo**  **(0.9% saline)**  **(*N* = 12)** |
| --- | --- | --- | --- |
| **Baseline Reproductive Hormones** |  |  |  |
|  | LH (IU/L) | 3.4 ± 0.4 | 3.5 ± 0.3 |
|  | FSH (IU/L) | 5.3 ± 0.4 | 5.8 ± 0.4 |
|  | Oestradiol (pmol/L) | 208.9 ± 25.5 | 197.8 ± 21.4 |

Mean ± SEM are presented. FSH, follicle-stimulating hormone; LH, luteinising hormone.

**Supplemental Table 4: Intranasal administration of kisspeptin stimulates reproductive hormone secretion in healthy women.**

| **Reproductive Hormone** | | **Intranasal Intervention** | **Mean**  **± SEM** | **Mean difference** | **95% CI** | ***P-*Value** |
| --- | --- | --- | --- | --- | --- | --- |
| **LH** |  |  |  |  |  |  |
|  | **AUC (h·IU/L)** |  |  |  |  |  |
|  |  | Placebo | -83.8 ± 22.3 | ---- | ---- | ---- |
|  |  | Kisspeptin 12.8 nmol/kg | 96.0 ± 45.8 | 179.8 | 89.2 to 270.5 | ***P* = 0.001**** |
|  | **Maximum increase from baseline (IU/L)** | |  |  |  |  |
|  |  | Placebo | 0.4 + 0.1 | ---- | ---- | ---- |
|  |  | Kisspeptin 12.8 nmol/kg | 1.4 ± 0.3 | 1.0 | 0.4 to 1.7 | ***P* = 0.004**** |
| **FSH** |  |  |  |  |  |  |
|  | **AUC (h·IU/L)** |  |  |  |  |  |
|  |  | Placebo | -112.5 ± 33.1 | ---- | ---- | ---- |
|  |  | Kisspeptin 12.8 nmol/kg | -36.1 ± 23.4 | 76.4 | 13.4 to 139.4 | ***P* = 0.022*** |
|  | **Maximum increase from baseline (IU/L)** | |  |  |  |  |
|  |  | Placebo | -0.1 ± 0.1 | ---- | ---- | ---- |
|  |  | Kisspeptin 12.8 nmol/kg | 0.3 ± 0.1 | 0.4 | 0.1 to 0.7 | ***P* = 0.019*** |

Mean (± SEM) area under the curve (AUC) of the change in serum luteinising hormone (LH) (h.IU/L), and follicle-stimulating hormone (FSH) (h.IU/L), and mean (± SEM) maximum increase from baseline in serum LH (IU/L), and FSH (IU/L). *P-*values represent comparison between the mean differences following intranasal kisspeptin administration vs. placebo. Groups were compared by paired t-tests. **P* < 0.05, ***P* < 0.01. *N* = 12.

**Supplemental Table 5: Baseline clinical characteristics at each study visit in patients with hypothalamic amenorrhoea.**

|  |  | **Kisspeptin**  **12.8 nmol/kg**  **(*N* = 10)** | **Placebo**  **(0.9% saline)**  **(*N* = 10)** |
| --- | --- | --- | --- |
| **Baseline Reproductive Hormones** |  |  |  |
|  | LH (IU/L) | 2.1 ± 0.5 | 2.3 ± 0.5 |
|  | FSH (IU/L) | 3.8 ± 0.5 | 4.1 ± 0.4 |
|  | Oestradiol (pmol/L) | 97.5 ± 6.6 | 114.1 ± 13.3 |

Mean ± SEM are presented. FSH, follicle stimulating hormone; LH, luteinising hormone.

**Supplemental Table 6: Intranasal administration of kisspeptin stimulates reproductive hormone secretion in patients with hypothalamic amenorrhoea.**

| **Reproductive Hormone** | | **Intranasal Intervention** | **Mean**  **± SEM** | **Mean difference** | **95% CI** | ***P-*Value** |
| --- | --- | --- | --- | --- | --- | --- |
| **LH** |  |  |  |  |  |  |
|  | **AUC (h·IU/L)** |  |  |  |  |  |
|  |  | Placebo | -156.3 ± 49.0 | ---- | ---- | ---- |
|  |  | Kisspeptin 12.8 nmol/kg | 600.6 ± 146.7 | 756.9 | 444.9 to 1069.0 | ***P* = < 0.001***** |
|  | **Maximum increase from baseline (IU/L)** | |  |  |  |  |
|  |  | Placebo | 0.1 ± 0.2 | ---- | ---- | ---- |
|  |  | Kisspeptin 12.8 nmol/kg | 4.4 ± 0.2 | 4.3 | 2.7 to 6.0 | ***P* < 0.001***** |
| **FSH** |  |  |  |  |  |  |
|  | **AUC (h·IU/L)** |  |  |  |  |  |
|  |  | Placebo | -104.7 ± 26.5 | ---- | ---- | ---- |
|  |  | Kisspeptin 12.8 nmol/kg | 474.9 ± 237.3 | 580.0 | 38.5 to 1121.0 | ***P* = 0.038*** |
|  | **Maximum increase from baseline (IU/L)** | |  |  |  |  |
|  |  | Placebo | 0.0 ± 0.1 | ---- | ---- | ---- |
|  |  | Kisspeptin 12.8 nmol/kg | 3.1 ± 0.3 | 3.1 | 0.1 to 6.1 | ***P* = 0.049*** |

Mean (± SEM) area under the curve (AUC) of the change in serum luteinising hormone (LH) (h.IU/L), and follicle-stimulating hormone (FSH) (h.IU/L), and mean (± SEM) maximum increase from baseline in serum LH (IU/L), and FSH (IU/L). *P-*values represent comparison between the mean differences following intranasal kisspeptin administration vs. placebo. Groups were compared by paired t-tests. **P* < 0.05, ****P* < 0.001. *N* = 10.

**Supplemental Table 7: Safety outcomes in healthy men.**

|  | | |  |  |  | |  | |  |
| --- | --- | --- | --- | --- | --- | --- | --- | --- | --- |
|  | | **Kisspeptin**  **3.2 nmol/kg**  **(*N* = 12)** | | **Kisspeptin**  **6.4 nmol/kg**  **(*N* = 12)** | | **Kisspeptin**  **12.8 nmol/kg**  **(*N* = 12)** | | **Kisspeptin**  **25.6 nmol/kg**  **(*N* = 12)** | **Placebo**  **(0.9% saline)**  **(*N* = 12)** |
| **Number of participants with at least 1 AE** | | 0 | | 0 | 0 | | 0 | | 0 |
| Cardiac disorders | Tachycardia | 0 | | 0 | 0 | | 0 | | 0 |
| Gastrointestinal disorders | Nausea  Vomiting  Abdominal pain  Diarrhoea | 0  0  0  0 | | 0  0  0  0 | 0  0  0  0 | | 0  0  0  0 | | 0  0  0  0 |
| General disorders | Procedural pain  Pyrexia  Chills  Hypotension  Hypertension  Decreased appetite | 0  0  0  0  0  0 | | 0  0  0  0  0  0 | 0  0  0  0  0  0 | | 0  0  0  0  0  0 | | 0  0  0  0  0  0 |
| Musculoskeletal disorders | Back pain  Arthralgia  Myalgia | 0  0  0 | | 0  0  0 | 0  0  0 | | 0  0  0 | | 0  0  0 |
| Nervous system disorders | Headache  Dizziness | 0  0 | | 0  0 | 0  0 | | 0  0 | | 0  0 |
| Respiratory disorders | Cough  Dyspnoea | 0  0 | | 0  0 | 0  0 | | 0  0 | | 0  0 |
| Skin disorders | Pruritus  Rash  Erythema | 0  0  0 | | 0  0  0 | 0  0  0 | | 0  0  0 | | 0  0  0 |
| **Number of participants with at least 1 SAE** | | 0 | | 0 | 0 | | 0 | | 0 |
| **AEs leading to discontinuation of study** | | 0 | | 0 | 0 | | 0 | | 0 |
| **Study-related AEs leading to discontinuation of study** | | 0 | | 0 | 0 | | 0 | | 0 |

Data represents the number of participants experiencing any adverse event (AE) or serious adverse event (SAE) and the number of participants experiencing any AE leading to discontinuation of study. *N* = 12.

**Supplemental Table 8: Safety outcomes in healthy women.**

|  | |  |  |
| --- | --- | --- | --- |
|  | | **Kisspeptin**  **12.8 nmol/kg**  **(*N* = 12)** | **Placebo**  **(0.9% saline)**  **(*N* = 12)** |
| **Number of participants with at least 1 AE** | | 0 | 0 |
| Cardiac disorders | Tachycardia | 0 | 0 |
| Gastrointestinal disorders | Nausea  Vomiting  Abdominal pain  Diarrhoea | 0  0  0  0 | 0  0  0  0 |
| General disorders | Procedural pain  Pyrexia  Chills  Hypotension  Hypertension  Decreased appetite | 0  0  0  0  0  0 | 0  0  0  0  0  0 |
| Musculoskeletal disorders | Back pain  Arthralgia  Myalgia | 0  0  0 | 0  0  0 |
| Nervous system disorders | Headache  Dizziness | 0  0 | 0  0 |
| Respiratory disorders | Cough  Dyspnoea | 0  0 | 0  0 |
| Skin disorders | Pruritus  Rash  Erythema | 0  0  0 | 0  0  0 |
| **Number of participants with at least 1 SAE** | | 0 | 0 |
| **AEs leading to discontinuation of study** | | 0 | 0 |
| **Study-related AEs leading to discontinuation of study** | | 0 | 0 |

Data represents the number of participants experiencing any adverse event (AE) or serious adverse event (SAE) and the number of participants experiencing any AE leading to discontinuation of study. *N* = 12.

**Supplemental Table 9: Safety outcomes in patients with hypothalamic amenorrhoea.**

|  | |  |  |
| --- | --- | --- | --- |
|  | | **Kisspeptin**  **12.8 nmol/kg**  **(*N* = 10)** | **Placebo**  **(0.9% saline)**  **(*N* = 10)** |
| **Number of participants with at least 1 AE** | | 0 | 0 |
| Cardiac disorders | Tachycardia | 0 | 0 |
| Gastrointestinal disorders | Nausea  Vomiting  Abdominal pain  Diarrhoea | 0  0  0  0 | 0  0  0  0 |
| General disorders | Procedural pain  Pyrexia  Chills  Hypotension  Hypertension  Decreased appetite | 0  0  0  0  0  0 | 0  0  0  0  0  0 |
| Musculoskeletal disorders | Back pain  Arthralgia  Myalgia | 0  0  0 | 0  0  0 |
| Nervous system disorders | Headache  Dizziness | 0  0 | 0  0 |
| Respiratory disorders | Cough  Dyspnoea | 0  0 | 0  0 |
| Skin disorders | Pruritus  Rash  Erythema | 0  0  0 | 0  0  0 |
| **Number of participants with at least 1 SAE** | | 0 | 0 |
| **AEs leading to discontinuation of study** | | 0 | 0 |
| **Study-related AEs leading to discontinuation of study** | | 0 | 0 |

Data represents the number of participants experiencing any adverse event (AE) or serious adverse event (SAE) and the number of participants experiencing any AE leading to discontinuation of study. *N* = 10.

**Supplemental Table 10: Intranasal administration of kisspeptin stimulates luteinising hormone release in adult male mice.**

| **Comparator** | | **Intranasal Intervention** | **Mean**  **± SEM** | **Mean difference** | **95% CI** | ***P-*Value** |
| --- | --- | --- | --- | --- | --- | --- |
| **AUC (h·ng/mL)** | |  |  |  |  |  |
|  | **Vs. placebo** |  |  |  |  |  |
|  |  | Placebo | 81.0 ± 8.2 | ---- | ---- | ---- |
|  |  | Kisspeptin 1 nM | 75.5 ± 6.3 | 5.5 | -59.0 to 48.0 | *P* = 0.834 |
|  |  | Kisspeptin 3 nM | 79.1 ± 5.8 | 2.0 | -55.5 to 51.6 | *P* = 0.941 |
|  |  | Kisspeptin 12.8 nM | 98.2 ± 15.5 | 17.2 | -34.1 to 68.4 | *P* = 0.498 |
|  |  | Kisspeptin 30 nM | 149.7 ± 13.3 | 68.7 | 19.2 to 118.2 | ***P* = 0.008**** |
|  |  | Kisspeptin 50 nM | 168.3 ± 38.5 | 87.3 | 33.8 to 140.8 | ***P* = 0.002**** |
|  |  |  |  |  |  |  |
|  | **Vs. kisspeptin 1 nM** | |  |  |  |  |
|  |  | Kisspeptin 1 nM | 75.5 ± 6.3 | ---- | ---- | ---- |
|  |  | Kisspeptin 3 nM | 79.1 ± 5.8 | 3.6 | -49.9 to 57.1 | *P* = 0.892 |
|  |  | Kisspeptin 12.8 nM | 98.2 ± 15.5 | 22.7 | -28.5 to 73.9 | *P* = 0.372 |
|  |  | Kisspeptin 30 nM | 149.7 ± 13.3 | 74.2 | 24.7 to 123.8 | ***P* = 0.005**** |
|  |  | Kisspeptin 50 nM | 168.3 ± 38.5 | 92.8 | 39.3 to 146.3 | ***P* = 0.001**** |
|  |  |  |  |  |  |  |
|  | **Vs. kisspeptin 3 nM** |  |  |  |  |  |
|  |  | Kisspeptin 3 nM | 79.1 ± 5.8 | ---- | ---- | ---- |
|  |  | Kisspeptin 12.8 nM | 98.2 ± 15.5 | 19.1 | -32.1 to 70.3 | *P* = 0.451 |
|  |  | Kisspeptin 30 nM | 149.7 ± 13.3 | 70.7 | 21.1 to 120.2 | ***P* = 0.007**** |
|  |  | Kisspeptin 50 nM | 168.3 ± 38.5 | 89.3 | 35.8 to 142.8 | ***P* = 0.002*** |
|  |  |  |  |  |  |  |
|  | **Vs. kisspeptin 12.8 nM** | |  |  |  |  |
|  |  | Kisspeptin 12.8 nM | 98.2 ± 15.5 | ---- | ---- | ---- |
|  |  | Kisspeptin 30 nM | 149.7 ± 13.3 | 51.6 | 4.5 to 98.6 | ***P* = 0.033*** |
|  |  | Kisspeptin 50 nM | 168.3 ± 38.5 | 70.2 | 18.9 to 121.4 | ***P* = 0.009**** |
|  |  |  |  |  |  |  |
|  | **Vs. kisspeptin 30 nM** |  |  |  |  |  |
|  |  | Kisspeptin 30 nM | 149.7 ± 13.3 | ---- | ---- | ---- |
|  |  | Kisspeptin 50 nM | 168.3 ± 38.5 | 18.6 | -30.9 to 68.1 | *P* = 0.448 |

Integrated LH response to kisspeptin-54 is represented by area under the curve (A.U.C.) measured in h·ng/mL at different doses of kisspeptin-54. Groups were compared by one-way ANOVA with Fisher’s LSD test (***P* < 0.01, **P* < 0.05). Mean ± SEM are presented. *N* = 5 per group except 12.8 nM where *N* = 6 and 30 nM where *N* = 7.
