# Supplementary material for: Intranasal kisspeptin administration rapidly stimulates gonadotropin release in humans
Source: eBioMedicine. 2025 Apr 11;115:105689. doi: 10.1016/j.ebiom.2025.105689 (PMC12018048; doi:10.1016/j.ebiom.2025.105689)
Supplement: Intranasal Kisspeptin Protocol [file mmc2.pdf]

Investigating the Effects of Intranasal Kisspeptin Administration on Reproductive Hormone Secretion

Version 1
23/09/2019

MAIN SPONSOR: Imperial College London

FUNDERS: NIHR, MRC and NIHR Imperial BRC
IRAS Project ID: 232585/93319
REC reference: 17/LO/1504,12/LO/0507

Protocol authorised by:

Name & Role	Date	Signature
Prof Waljit Dhillo (PI + CI for ICL site)	23/09/2019	[signature]
Dr Alexander Comninos (CI for ICHNT site)	23/09/2019	[signature]

© Imperial College of Science, Technology and Medicine

Study Management Group

Chief Investigators: Dr Alexander N Comninos & Professor Waljit S Dhillo

Co-investigators: Dr Edouard Mills

Study Management: Dr Edouard Mills

Study Coordination Centre

For general queries, supply of study documentation, and collection of data, please contact:

Study Coordinator: Dr Edouard Mills
Address: Section of Endocrinology & Investigative Medicine, 6th Floor Commonwealth Building, Imperial College London, Hammersmith Hospital Campus, Du Cane Road, London, W12 0NN.
Tel: 0207 594 3487 ***E-mail:*** e.mills@imperial.ac.uk

Clinical Queries

Clinical queries should be directed to **Dr Edouard Mills** who will direct the query to the appropriate person.

Sponsor

Imperial College London is the main research Sponsor for this study. For further information regarding the sponsorship conditions, please contact the Head of Regulatory Compliance at:

Research Governance and Integrity Team
Imperial College London and Imperial College Healthcare NHS Trust
Room 215, Level 2, Medical School Building
Norfolk Place
London, W2 1PG
Tel: **0207 594 1862**

© Imperial College of Science, Technology and Medicine

Funder

This study will be funded from Professor Waljit Dhillo's NIHR Research Professorship and Senior Investigator Award.

This protocol describes the '**Investigating the Effects of Intranasal Kisspeptin Administration on Reproductive Hormone Secretion'** study and provides information about procedures for entering participants. Every care was taken in its drafting, but corrections or amendments may be necessary. These will be circulated to investigators in the study. Problems relating to this study should be referred, in the first instance, to the Chief Investigators.

This study will adhere to the principles outlined in the UK Policy Frame Work for Health and Social Care Research. It will be conducted in compliance with the protocol, the Data Protection Act and other regulatory requirements as appropriate.

Table of Contents — Page No

1. INTRODUCTION 6
1.1. BACKGROUND 6
1.2. RATIONALE FOR CURRENT STUDY 6
2. STUDY OBJECTIVES 6
3. STUDY DESIGN 7
4. PARTICIPANT ENTRY 10
4.1. PRE-REGISTRATION EVALUATIONS 10
4.2. INCLUSION AND EXCLUSION CRITERIA 11
INCLUSION CRITERIA 11
EXCLUSION CRITERIA 11
4.3. WITHDRAWAL CRITERIA 12
5. ADVERSE EVENTS 12
5.1. DEFINITIONS 12
5.2. REPORTING PROCEDURES 13
6. ASSESSMENT AND FOLLOW-UP 13
7. STATISTICS AND DATA ANALYSIS 14
8. REGULATORY ISSUES 14
8.1. ETHICS APPROVAL 14
8.2. CONSENT 14
8.3. CONFIDENTIALITY 15
8.4. INDEMNITY 15
8.5. SPONSOR 15
8.6. FUNDING 15
8.7. AUDITS 15
9. STUDY MANAGEMENT 15
10. PUBLICATION POLICY 15
11. REFERENCES 16

© Imperial College of Science, Technology and Medicine

GLOSSARY OF ABBREVIATIONS

BP	Blood pressure
FSH	Follicle stimulating hormone
GnRH	Gonadotropin releasing hormone
HA	Hypothalamic amenorrhoea
HPG	Hypothalamic-pituitary-gonadal
HR	Heart rate
IN	Intranasal
KP54	Kisspeptin-54
LH	Luteinising hormone

© Imperial College of Science, Technology and Medicine

KEYWORDS
Kisspeptin, fertility, neuropeptides, reproduction, neuroendocrinology, intranasal.

STUDY SUMMARY

**TITLE**	Investigating the Effects of Intranasal Kisspeptin Administration on Reproductive Hormone Secretion.
**DESIGN**	Randomised, double-blind, placebo-controlled, crossover study comparing intranasal kisspeptin administration versus intranasal placebo on reproductive hormone release in humans.
**AIMS**	To investigate the effects of intranasal kisspeptin administration on reproductive hormone release in healthy volunteers (men *and* women) and female patients with reproductive disorders (including hypothalamic amenorrhoea).
**OUTCOME MEASURES**	Blood levels of reproductive hormones measured using automated chemiluminescent immunoassays at baseline and every 15 minutes for 4 hours. Safety monitoring: heart rate, blood pressure, and the presence of adverse symptoms measured using automated blood pressure monitor every 15 minutes for 4 hours
**POPULATION**	A minimum of 12 healthy volunteers (i.e., healthy men and healthy women) and 6 patients with reproductive disorders.
**ELIGIBILITY**	Healthy volunteers (with normal reproductive function [men and women], including regular menstrual cycles and not taking hormonal contraception [women]. Patients with reproductive disorders, diagnosed in accordance with established guidelines (e.g. Endocrine Society guidelines for hypothalamic amenorrhoea).
**DURATION**	Recruitment will stop when sufficient participant numbers have been enrolled to ensure the planned sample size has been achieved. The study will stop once all participants have completed the study protocol.

© Imperial College of Science, Technology and Medicine

1. INTRODUCTION

1.1. BACKGROUND

Reproductive disorders encompass a broad range of highly prevalent conditions, frequently caused by aberrations in the hypothalamic-pituitary-gonadal (HPG) reproductive axis. Indeed, recent epidemiological data highlights that infertility is experienced by 12.5% of women and 10.1% of men in the United Kingdom (1). Despite the major health burden associated with reproductive disorders, many standard therapies are limited by poor efficacy, contraindicated in many, and carry significant side-effects. Hence, novel, safe, and effective clinical strategies are therefore much-needed.

The reproductive neuropeptide kisspeptin has emerged as critical for mammalian reproduction due to its key role as an upstream regulator of gonadotropin-releasing hormone (GnRH) secretion. This in turn controls downstream gonadal function via the gonadotropins luteinising hormone (LH) and follicle-stimulating hormone (FSH). As such, due to kisspeptin's key role in regulating physiological reproductive hormone secretion, there has been accumulating clinical and research interest in using kisspeptin-based medicines to restore hormonal secretion in common reproductive disorders, including hypothalamic amenorrhea (HA) (2–4), hyperprolactinemia (5, 6), type 2 diabetes and obesity-related hypogonadism (7), and as a safer trigger for inducing oocyte maturation in *in vitro* fertilization (8–10).

However, current administration of kisspeptin is confined to the invasive subcutaneous or intravenous routes which limits patient acceptability and clinical use. Therefore, alternative delivery routes could overcome this and further accelerate the development of kisspeptin-based therapeutics in humans. To this end, the aim of this study is to investigate the effects of intranasal kisspeptin administration on reproductive hormone release in humans.

1.2. RATIONALE FOR CURRENT STUDY

This study is designed to investigate the effects of intranasal kisspeptin administration on reproductive hormone release in healthy volunteers (men and women) and female patients with reproductive disorders (such as HA). Participants will attend for at least two study visits each comparing intranasal kisspeptin administration versus intranasal placebo, as part of a randomised, double-blind, placebo-controlled, crossover study. Following self-administration of intranasal kisspeptin or placebo (using a nasal spray device), serum reproductive hormones will be measured every 15 minutes for 4 hours.

Primary Hypothesis: Intranasal kisspeptin administration stimulates reproductive hormone secretion in healthy volunteers and patients with common reproductive disorders.

2. STUDY OBJECTIVES

Primary Outcome:
Change in serum LH following intranasal administration of kisspeptin compared to placebo. Blood samples will be taken at baseline (pre-administration) and every 15 minutes for 4 hours.

© Imperial College of Science, Technology and Medicine

Secondary Outcomes:
1. Change in serum FSH following intranasal administration of kisspeptin compared to placebo. Blood samples will be taken at baseline (pre-administration) and every 15 minutes for 4 hours.
2. [Men]: Change in serum testosterone following intranasal administration of kisspeptin compared to placebo. Measurements will be taken at baseline (pre-administration) and every 15 minutes for 4 hours.
3. [Women]: Change in serum oestradiol following intranasal administration of kisspeptin compared to placebo. Measurements will be taken at baseline (pre-administration) and every 15 minutes for 4 hours.
4. [Women]: Change in serum progesterone following intranasal administration of kisspeptin compared to placebo. Measurements will be taken at baseline (pre-administration) and every 15 minutes for 4 hours.
5. Safety assessments: standard adverse event reporting, blood pressure, and heart rate recordings.

3. STUDY DESIGN

This is a randomised, double-blind, placebo-controlled, crossover study investigating the effects of intranasal kisspeptin administration on reproductive hormone release in healthy volunteers (men and women) and patients with reproductive disorders. The cross-over design has been chosen as it is the most rigorous method to determine a treatment effect, by minimising the effect of confounding variables. Each participant will be required to attend all study visits, hence serving as their own control.

The study will recruit three groups of participants. *Group A* will consist of healthy men with normal reproductive function and will also determine the optimal dose for intranasal kisspeptin administration. *Group B* will consist of healthy (ovulatory) women with normal reproductive function. *Group C* will consist of female patients with reduced reproductive function, such as HA. The groups will not be recruited in parallel, as the results from *Group A* will determine the optimal dosing for *Groups B* and *Groups C*.

Participants
A minimum of 12 healthy volunteers (*Group A* and *Group B*) and 6 patients with reproductive disorders (*Group C*) will be recruited (described in Section 4).

Pre-Study Visits
Recruited participants will be asked to abstain from alcohol, caffeine, and sexual activities from 22:00 before their study visits and to consume a normal breakfast on their study days. All study visits will start in the morning to control for circadian hormonal changes. Study visits for participants in *Group A* and participants in *Group C* (e.g., anovulatory women) will take place at least 1 week apart (to ensure washout). Study visits for participants in *Group B* will take place during the follicular phase of the menstrual cycle, i.e., days 2-10 inclusive (to control for reproductive hormone changes over the menstrual cycle).

Study Visits
Study visits will take place at the Clinical Research Unit (Imperial College Healthcare NHS Trust) and all participants within each group will receive the same protocol during each study visit.

© Imperial College of Science, Technology and Medicine

Group A (Healthy Men): Kisspeptin has not been administered via the intranasal route to humans and thus this part of the study will also determine the optimal dosing protocol for intranasal kisspeptin delivery. Participants will attend for five study visits each and will receive the following five interventions (on separate visits and in random order) via the intranasal route: 3.2, 6.4, 12.8, and 25.6 nmol/kg of kisspeptin-54, and 0.9% saline (placebo). After self-administration of kisspeptin-54 or placebo at timepoint 0 minutes, serum levels of LH, FSH, and testosterone will be measured every 15 minutes for 4 hours ***(see Figure 1)***.

Figure 1 – Protocol Schematic (Group A)

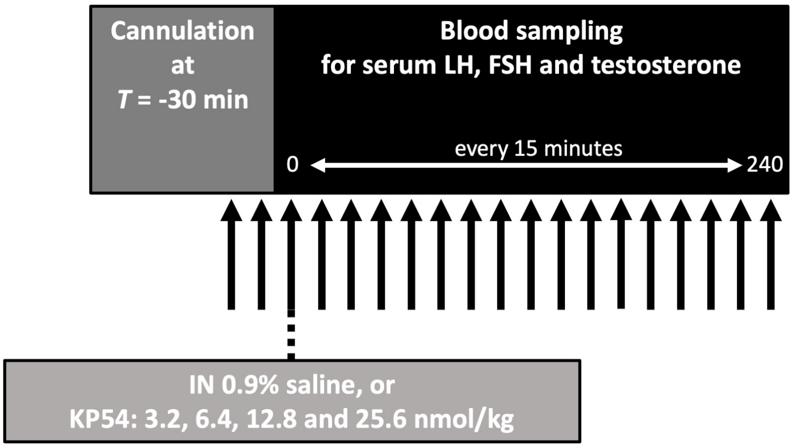

Group B (Healthy Women): Participants will attend for two study visits each, receiving kisspeptin-54 and 0.9% saline (placebo) (on separate visits and in random order) via the intranasal route. The dose of kisspeptin-54 will be determined based on the results of *Group A*, i.e., the dose of kisspeptin-54 which elicits the greatest maximal increase in serum LH in healthy men. After self-administration of kisspeptin-54 or placebo at timepoint 0 minutes, serum levels of LH, FSH, oestradiol, and progesterone will be measured every 15 minutes for 4 hours ***(see Figure 2)***.

Figure 2 – Protocol Schematic (Group B)

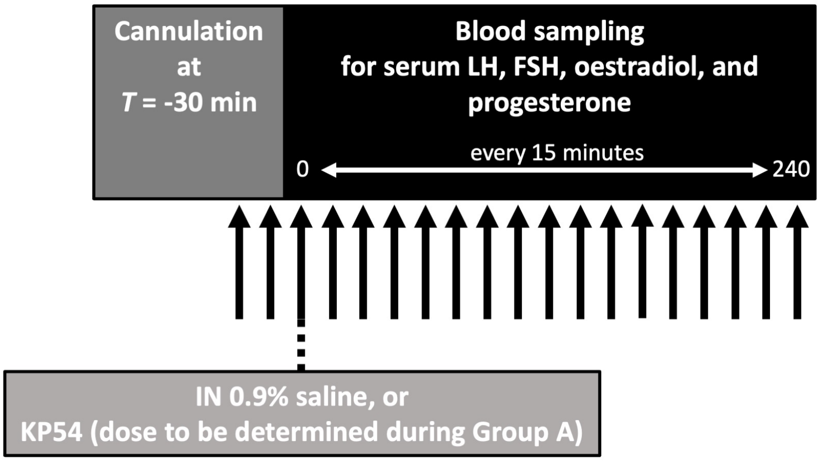

Group C (Female patients with Reproductive Disorders): Patients will attend for two study visits each, receiving kisspeptin-54 and 0.9% saline (placebo) (on separate visits and in

© Imperial College of Science, Technology and Medicine

random order) via the intranasal route. The dose of kisspeptin-54 will be determined based on the results of *Group A (*described above) and will be the same dose examined in *Group B*. After self-administration of kisspeptin-54 or placebo at timepoint 0 minutes, serum levels of LH, FSH, oestradiol, and progesterone will be measured every 15 minutes for 4 hours ***(see Figure 3)***.

Figure 3 – Protocol Schematic (Group C)

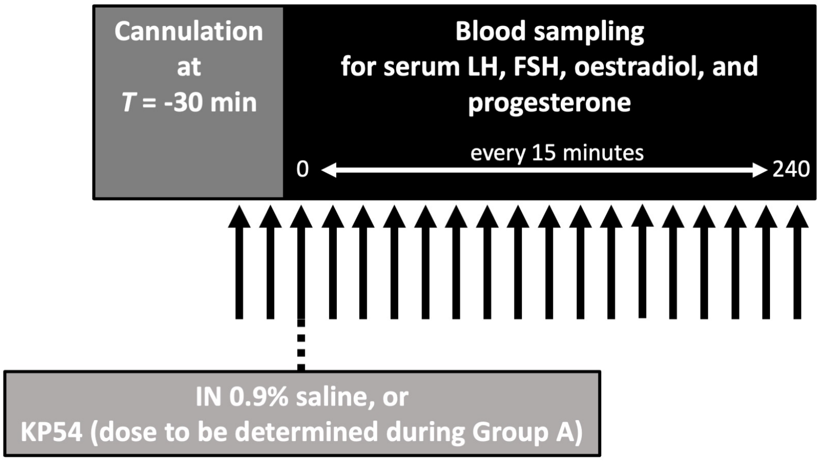

<u>*For all groups*</u>: On arrival to the Clinical Research Unit and following a period of acclimatisation, an intravenous cannula will be inserted into one antecubital fossa (for blood sampling). Female participants will have their urine tested for ß-hCG (pregnancy test) at each study visit to ensure that they are not pregnant. Next, participants will receive a standardised education session explaining the procedure of intranasal administration (to ensure good technique). Blood will then be sampled at timepoints -30, -15, and 0 minutes (pre-administration baseline), followed by intranasal administration of kisspeptin-54 or 0.9% saline (placebo).

Thereafter, serum levels of LH, FSH, and testosterone (*Group A*), or oestradiol and progesterone (*Group B* and *Group C*) will be measured every 15 minutes for 4 hours. Heart rate, blood pressure, and the presence of adverse symptoms will also be recorded every 15 minutes. At least two clinically trained investigators will be present throughout each study visit.

Participants, study visit investigators, and the data analysts will be blinded to the identity of kisspeptin or placebo. The order of study visits will be randomised by an independent investigator prior to recruitment of the first participant. The randomisation sequence will be held centrally at Imperial College London by the CI's (Dr Alexander Comninos and Professor Waljit Dhillo) who are not involved in the study visits and data analysis.

Safety of participants during the Study
Participants will remain in the investigation unit for a short period of observation after termination of the study visit. At least two clinically trained investigators will be present throughout each study visit. During the study, blood pressure, and heart rate measurements will be taken at 15-minute intervals. Participants will be encouraged to report any unusual or unpleasant sensation to the investigators immediately. Any significant adverse effects will lead to withdrawal of the individual and any serious adverse effects will terminate the whole study.

© Imperial College of Science, Technology and Medicine

Throughout the study there will be at least one physician available 24 hours per day via a direct line, with a second physician on back up, and a secondary direct line to one of the senior physicians. Although we do not anticipate any serious adverse effects, participants will be provided with contact numbers and clear instructions that, if they feel unwell, they should call us. Kisspeptin is rapidly cleared (11, 12) and participants would not be anticipated to experience any delayed effects on completion of the study visit.

The Chief Investigators, Dr Alexander Comninos and Professor Waljit Dhillo, are on site, and any potential side−effects of the study will be reviewed regularly by Dr Comninos and Professor Dhillo and the other main investigator for the study (Dr Edouard Mills). The safety of the participants is of the highest importance throughout the study. An internal audit will be completed at the end of the study. Study efficacy and safety will be regularly reviewed in fortnightly meetings. A formal data monitoring committee is not necessary as kisspeptin has been given to over 500 human subjects and is safe and well tolerated. The study may be subject to audit by Imperial College London under their remit as sponsor and other regulatory bodies to ensure adherence to GCP and the UK Policy Framework for Health and Social Care Research.

Kisspeptin
Kisspeptin is a naturally occurring and safe hormone, which is known to play a critical physiological role in human fertility (13, 14). Kisspeptin has been given safely to over 500 male and female participants in subcutaneous and intravenous form without side effects (15–18). Levels of kisspeptin in the circulation increase ~7,000-fold during normal pregnancy without any known adverse effects (19). We do not anticipate any adverse effects of the doses of kisspeptin proposed in the study, however if any serious adverse effects are noted at any time, we will immediately suspend the study, report to the sponsor and review continuation of the research with the research ethics committee. The intravenous half-life of kisspeptin is short (28 minutes) and if any unexpected adverse effects are encountered (not expected), we would expect these to resolve rapidly with supportive management.

Kisspeptin-54 is stored at -20°C as per manufacturer's guidelines. Freeze-dried kisspeptin-54 vials will be reconstituted on the morning of each study visit to produce a solution containing the target dose, which will be self-administered by the participant (under supervision of the clinical team) using a nasal spray device

The maximum doses of kisspeptin-54 administered will be 25.6nmol/kg. We have already administered these identical doses during our previous studies (8, 15, 16, 20) that have demonstrated to safely produce a biological effect without untoward side effects. Intranasal placebo administration will consist of saline (identical in volume and appearance to kisspeptin), thus respecting the study's double blinded design.

4. PARTICIPANT ENTRY

4.1. PRE-REGISTRATION EVALUATIONS

We will recruit individuals with intact and reduced/absent fertility who are aged ≥18 years old. We will advertise via print (including posters around university campuses, local and regional newspapers and magazines) and online advertisements, including social media, as well as collaborate with the Endocrinology and Reproductive Medicine Departments at Imperial College Healthcare NHS Trust to identify potential participants from clinics. Upon first contact,

© Imperial College of Science, Technology and Medicine

potential participants will be provided with a Participant Information Sheet and a self-report questionnaire (returned to a secure and dedicated email address: imperial.kisspeptin@nhs.net), to identify any objective exclusion criteria. If this does not identify any objective exclusion criteria the individual will be invited to a 1-hour face-to-face screening visit, which will be carried out by an experienced Research Doctor or Research Nurse. The face-to-face screening visit will involve going through the details of the study, obtaining consent, taking relevant medical history, clinical examination, vital signs assessment, an ECG, and clinical laboratory testing.

During the screening visit, the study will be explained again in full, and the participant will be given the opportunity to ask any further questions they may have. Informed written consent will be taken before proceeding to any further assessment. Participants will be informed that they are free to withdraw from the study at any time.

Blood samples will be collected for investigations including reproductive hormone levels, haematology, and biochemistry, if the participant consents.

Any new diagnoses that are made will be discussed with the participant by one of the study doctors and if the participant consents, their GP will be contacted to arrange further management or specialist referral as appropriate.

4.2. INCLUSION AND EXCLUSION CRITERIA

INCLUSION CRITERIA

Healthy volunteers *(Group A* and *Group B)*

1. Aged 18–70 years
2. Non-smokers
3. Free of current or past physical or psychiatric illness
4. Naive to psychoactive substances, prescribed or illicit, for a minimum of 6 months prior to screening.
5. Regular menstrual cycles (women)

For female patients with common reproductive disorders *(Group C)*

1. As above *PLUS:*
2. Patients diagnosed in accordance with established guidelines (e.g. Endocrine Society guidelines for hypothalamic amenorrhoea)

EXCLUSION CRITERIA

1. History of any medical, psychological or other condition, or use of any medications, including over-the-counter products and hormonal therapies, which, in the opinion of the investigators, would either interfere with the study or potentially cause harm to the participant
2. Medical or psychological conditions that would impair their ability to participate reliably in the study or give informed consent
3. Pregnancy and/or breastfeeding

© Imperial College of Science, Technology and Medicine

4. Without access at home to a telephone, or other factor likely to interfere with ability to participate reliably in the study
5. History of hypersensitivity to any of the components administered
6. Treatment with an investigational drug within the preceding 2 months
7. Those who have or intend to donate blood or blood products within three months before or following study completion
8. A history of major haematological, renal, thyroid or hepatic abnormalities or significant cardiovascular disease
9. A history of cancer

4.3. WITHDRAWAL CRITERIA

If a participant loses capacity during the study, then they would be withdrawn from further participation in the study. However, any data or samples that we had already collected when they were able to give consent would be used in the study. We will seek their informed written consent for this at the screening visit. If consent is not given to this and the participant loses capacity, samples would be disposed of in accordance with the Human Tissue Authority's Code of Practice following completion of the study and not kept for use in future ethically approved research.

Any significant adverse effects will lead to withdrawal of the individual and any serious adverse effects will terminate the whole study.

It will be made clear to participants that they will be free to withdraw from the study at any time without providing any reason.

5. ADVERSE EVENTS

5.1. DEFINITIONS

Adverse Event (AE): any untoward medical occurrence in a patient or clinical study subject.

Serious Adverse Event (SAE): any untoward medical occurrence or effect that:

- **Results in death**
- **Is life-threatening** – *refers to an event in which the subject was at risk of death at the time of the event; it does not refer to an event which hypothetically might have caused death if it were more severe*
- **Requires hospitalisation, or prolongation of existing inpatients' hospitalisation**
- **Results in persistent or significant disability or incapacity**
- **Is a congenital anomaly or birth defect**

Medical judgement should be exercised in deciding whether an AE is serious in other situations. Important AEs that are not immediately life-threatening or do not result in death or hospitalisation but may jeopardise the subject or may require intervention to prevent one of the other outcomes listed in the definition above, should also be considered serious.

© Imperial College of Science, Technology and Medicine

5.2. REPORTING PROCEDURES

All adverse events should be reported. Depending on the nature of the event the reporting procedures below should be followed. Any questions concerning adverse event reporting should be directed to the Chief Investigators in the first instance.

5.3.1 Non serious AEs

All such events, whether expected or not, should be recorded.

5.3.2 Serious AEs

An SAE form should be completed and emailed to the Chief Investigators within 24 hours. However, hospitalisations for elective treatment of a pre-existing condition do not need reporting as SAEs.

All SAEs should be reported to the Riverside Research Ethics Committee (studies in men) and the West London Research Ethics Committee (studies in women) where in the opinion of the Chief Investigators, the event was:

- 'related', ie resulted from the administration of any of the research procedures; and
- 'unexpected', ie an event that is not listed in the protocol as an expected occurrence

Reports of related and unexpected SAEs should be submitted within 15 days of the Chief Investigators becoming aware of the event, using the NRES SAE form for non-IMP studies. The Chief Investigators must also notify the Sponsor of all related and unexpected SAEs.

Local investigators should report any SAEs as required by their Local Research Ethics Committee, Sponsor and/or Research & Development Office.

Contact details for reporting SAEs
RGIT@imperial.ac.uk
CI email (and contact details below)
Please send SAE forms to: w.dhillo@imperial.ac.uk a.comninos@imperial.ac.uk
Tel: 0207 594 3487 (Mon to Fri 09.00 – 17.00)

6. ASSESSMENT AND FOLLOW-UP

No specific follow-up will be required.

Very rarely, abnormalities may be detected (including during the screening visit) that warrant further investigation. In this situation the result would be communicated first to the participant and then to the participant's GP, who will arrange the appropriate specialist referral. A significant abnormality (or change in eligibility) would lead to exclusion from further participation in the study.

The definition of the end of the study will be once the last participant has completed their final study visit and the data collection (which includes analysis of blood samples) is complete.

© Imperial College of Science, Technology and Medicine

7. STATISTICS AND DATA ANALYSIS

This will be the first study investigating the effects of intranasal kisspeptin administration on reproductive hormone release in humans. Our previous work in healthy volunteers demonstrates that intravenous kisspeptin increases serum LH (mean 10.2 IU/L and standard deviation 0.4 IU/L), compared with placebo administration (mean 2.0 IU/L, standard deviation 0.1 IU/L) (11), and we predict a similar response following intranasal administration. Therefore, using these data, with $\alpha = 0.05$, power = 0.8 and effect size = 0.997, we performed a power calculation resulting in a sample size of 10 participants per group (*Group A* and *Group B*). To allow for dropouts and exclusions (estimated to be around 20%), 12 participants per group (*Group A* and *Group B*) will be recruited. It should be noted that regarding patients with reproductive disorders (*Group C*), the sample size will depend upon the specific disorder. For instance, our previous work demonstrates that the gonadotropin response following kisspeptin administration is similar in patients with polycystic ovary syndrome versus healthy women but augmented in patients with hypothalamic amenorrhoea [unpublished data].

Hormone level data will be assessed for normality and appropriate statistical methods chosen. Continuous data will be reported as measures of central tendency and of spread (e.g., mean and standard error of the mean or median and interquartile range as appropriate). For all study groups, time profiles of hormone levels during the 4-hour study period will be compared using two-way ANOVA with Bonferroni multiple comparison test [if the data is parametric], or Kruskal-Wallis test [if the data is non-parametric]. Mean responses (e.g., maximal increase and area under curve) will be compared using one-way ANOVA with Bonferroni multiple comparison test (*Group A*) and paired t-tests (*Group B* and *Group C*) [if the data is parametric] or Kruskal-Wallis test (*Group A*) and Wilcoxon signed-rank test (*Group B* and *Group C*) [if the data is non- parametric]. $P < 0.05$ will be considered statistically significant.

Data and all appropriate documentation will be stored for a minimum of 10 years after the completion of the study, including the follow-up period.

8. REGULATORY ISSUES

8.1. ETHICS APPROVAL

The Study Coordination Centre has obtained approval from the Riverside Research Ethics Committee, London, UK (ref: 17/LO/1504) [studies in men] and the West London Research Ethics Committee, London, UK (ref: 12/LO/0507) [studies in women] and Health Research Authority (HRA). The study must also receive confirmation of capacity and capability from each participating NHS Trust before accepting participants into the study or any research activity is carried out. The study will be conducted in accordance with the recommendations for physicians involved in research on human subjects adopted by the 18th World Medical Assembly, Helsinki 1964 and later revisions.

8.2. CONSENT

Consent to enter the study must be sought from each participant only after a full explanation has been given, an information leaflet offered, and time allowed for consideration. Signed participant consent should be obtained. The right of the participant to refuse to participate without giving reasons must be respected. After the participant has entered the study the clinician remains free to give alternative treatment to that specified in the protocol at any stage if he/she feels it is in the participant's best interest, but the reasons for doing so should be recorded. In these cases, the participants remain within the study for the purposes of follow-

© Imperial College of Science, Technology and Medicine

up and data analysis. All participants are free to withdraw at any time from the protocol treatment without giving reasons and without prejudicing further treatment.

8.3. CONFIDENTIALITY

The Chief Investigators will preserve the confidentiality of participants taking part in the study and is registered under the Data Protection Act. Data will be pseudonymised. Data will not be transferred to a third party.

The results are likely to be published in the six months following the study. The results might be reported in internal reports, peer reviewed scientific journals, other publications or as part of conference presentations. Confidentiality will be ensured at all times, and participants will not be identified in any publication. At the end of the study, the results of the study can be made available to the participant, or their GP should they wish.

8.4. INDEMNITY

Imperial College London holds negligent harm and non-negligent harm insurance policies which apply to this study.

8.5. SPONSOR

Imperial College London will act as the main Sponsor for this study. Delegated responsibilities will be assigned to the NHS trusts taking part in this study.

8.6. FUNDING

This study will be funded from Professor Waljit Dhillo’s NIHR Research Professorship and Senior Investigator Award. Participants will receive £100 per study visit on completion of the study to cover expenses including travel costs, time off work and lost earnings. The investigators do not receive any payment for this study.

8.7. AUDITS

The study may be subject to audit by Imperial College London/ Imperial College Healthcare NHS Trust under their remit as sponsor and other regulatory bodies to ensure adherence to GCP and the UK Policy Frame Work for Health and Social Care Research.

9. STUDY MANAGEMENT

The day-to-day management of the study will be led by Dr Edouard Mills, under the supervision of the Chief Investigators (Dr Alexander N Comninos and Professor Waljit S Dhillo).

10. PUBLICATION POLICY

We aim to disseminate data generated during the study via publication in peer reviewed medical journals, presentation at conferences and publication in the lay press as appropriate. During publication the data will be completely anonymised and no personal information will be published. Participant confidentiality will be maintained throughout. Participants and their GPs (with the participant’s consent) will be provided with a copy of published data should they wish.

© Imperial College of Science, Technology and Medicine

11. REFERENCES

1. Datta J, et al. Prevalence of Infertility and Help Seeking Among 15 000 Women and Men. *Human Reproduction*. 2016;31(9):2108–18.
2. Jayasena CN, et al. Subcutaneous Injection of Kisspeptin-54 Acutely Stimulates Gonadotropin Secretion in Women with Hypothalamic Amenorrhea, But Chronic Administration Causes Tachyphylaxis. *J Clin Endocrinol Metab*. 2009;94(11):4315–4323.
3. Jayasena CN, et al. Twice-weekly Administration of kisspeptin-54 for 8 Weeks Stimulates Release of Reproductive Hormones in Women With Hypothalamic Amenorrhea. *Clin Pharmacol Ther*. 2010;88(6):840–847.
4. Jayasena CN, et al. Increasing LH Pulsatility in Women With Hypothalamic Amenorrhoea Using Intravenous Infusion of Kisspeptin-54. *Journal of Clinical Endocrinology and Metabolism*. 2014;99(6):e953-61.
5. Sonigo C, et al. Hyperprolactinemia-induced Ovarian Acyclicity Is Reversed by Kisspeptin Administration. *Journal of Clinical Investigation*. 2012;122(10):3791–5.
6. Millar RP, et al. Hypothalamic-Pituitary-Ovarian Axis Reactivation by Kisspeptin-10 in Hyperprolactinemic Women With Chronic Amenorrhea. *J Endocr Soc*. 2017;1(11):1362–1371.
7. George JT, et al. Exploring the Pathophysiology of Hypogonadism in Men With Type 2 Diabetes: Kisspeptin-10 Stimulates Serum Testosterone and LH Secretion in Men With Type 2 Diabetes and Mild Biochemical Hypogonadism. *Clin Endocrinol (Oxf)*. 2013;79(1):100–104.
8. Jayasena CN, et al. Kisspeptin-54 Triggers Egg Maturation in Women Undergoing In Vitro Fertilization. *Journal of Clinical Investigation*. 2014;124(8):3667–3677.
9. Abbara A, et al. Efficacy of Kisspeptin-54 to Trigger Oocyte Maturation in Women at High Risk of Ovarian Hyperstimulation Syndrome (OHSS) During In Vitro Fertilization (IVF) Therapy. *Journal of Clinical Endocrinology and Metabolism*. 2015;100(9):3322–3331.
10. Abbara A, et al. A Second Dose of kisspeptin-54 Improves Oocyte Maturation in Women at High Risk of Ovarian Hyperstimulation Syndrome: A Phase 2 Randomized Controlled Trial. *Human Reproduction*. 2017;32(9):1915–1924.
11. Dhillo WS, et al. Kisspeptin-54 Stimulates the Hypothalamic-Pituitary Gonadal Axis in Human Males. *J Clin Endocrinol Metab*. 2005;90(12):6609–6615.
12. Jayasena CN, et al. The Effects of Kisspeptin-10 on Reproductive Hormone Release Show Sexual Dimorphism in Humans. *J Clin Endocrinol Metab*. 2011;96(12):E1963–E1972.
13. De Roux N, et al. Hypogonadotropic hypogonadism due to loss of function of the KiSS1-derived peptide receptor GPR54. *Proc Natl Acad Sci U S A*. 2003;100(19):10972–10976.
14. Seminara SB, et al. The GPR54 gene as a regulator of puberty. *N Engl J Med*. 2003;349(17):1614–1627.
15. Dhillo WS, et al. Kisspeptin-54 stimulates the hypothalamic-pituitary gonadal axis in human males. *J Clin Endocrinol Metab*. 2005;90(12):6609–15.
16. Dhillo WS, et al. Kisspeptin-54 stimulates gonadotropin release most potently during the preovulatory phase of the menstrual cycle in women. *Journal of Clinical Endocrinology and Metabolism*. 2007;92(10):3958–3966.
17. Jayasena CN, et al. A single injection of kisspeptin-54 temporarily increases luteinizing hormone pulsatility in healthy women. *Clin Endocrinol (Oxf)*. 2013;79(4):558–563.
18. Jayasena CN, et al. Increasing LH pulsatility in women with hypothalamic amenorrhoea using intravenous infusion of kisspeptin-54. *Journal of Clinical Endocrinology and Metabolism*. 2014;99(6):953–961.
19. Jayasena CN, et al. The identification of elevated urinary kisspeptin-immunoreactivity during pregnancy. *Ann Clin Biochem*. 2015;52(Pt 3):395–8.
20. Jayasena CN, et al. The Effects of Kisspeptin-10 on Reproductive Hormone Release Show Sexual Dimorphism in Humans. *J Clin Endocrinol Metab*. 2011;96(12):E1963–E1972.

© Imperial College of Science, Technology and Medicine
